# Pre- and Postnatal Arsenic Exposure and Body Size to 2 Years of Age: A Cohort Study in Rural Bangladesh

**DOI:** 10.1289/ehp.1003378

**Published:** 2012-04-13

**Authors:** Kuntal K Saha, Annette Engström, Jena Derakhshani Hamadani, Fahmida Tofail, Kathleen M Rasmussen, Marie Vahter

**Affiliations:** 1 International Centre for Diarrhoeal Disease Research, Bangladesh, Dhaka, Bangladesh; 2 International Food Policy Research Institute, Washington, DC, USA; 3 Institute of Environmental Medicine, Karolinska Institutet, Stockholm, Sweden; 4 Division of Nutritional Sciences, Cornell University, Ithaca, New York, USA

**Keywords:** arsenic exposure, Bangladesh, child growth, drinking water, maternal urine, MINIMat, pregnancy

## Abstract

Background: Exposure to arsenic via drinking water has been associated with adverse pregnancy outcomes and infant morbidity and mortality. Little is known, however, about the effects of arsenic on child growth.

Objective: We assessed potential effects of early-life arsenic exposure on weight and length of children from birth to 2 years of age.

Methods: We followed 2,372 infants born in a population-based intervention trial in rural Bangladesh. Exposure was assessed by arsenic concentrations in urine (U-As) of mothers (gestational weeks 8 and 30) and children (18 months old). Child anthropometry was measured monthly in the first year and quarterly in the second. Linear regression models were used to examine associations of U-As (by quintiles) with child weight and length, adjusted for age, maternal body mass index, socioeconomic status, and sex (or stratified by sex).

Results: Median (10th–90th percentiles) U-As concentrations were about 80 (25–400) µg/L in the mothers and 34 (12–159) µg/L in the children. Inverse associations of maternal U-As with child’s attained weight and length at 3–24 months were markedly attenuated after adjustment. However, associations of U-As at 18 months with weight and length at 18–24 months were more robust, particularly in girls. Compared with girls in the first quintile of U-As (< 16 µg/L), those in the fourth quintile (26–46 µg/L) were almost 300 g lighter and 0.7 cm shorter, and had adjusted odds ratios (95% confidence interval) for underweight and stunting of 1.57 (1.02–2.40) and 1.58 (1.05–2.37), respectively, at 21 months.

Conclusions: Postnatal arsenic exposure was associated with lower body weight and length among girls, but not boys.

Inorganic arsenic is found in drinking water based on groundwater in many countries, including Bangladesh. Chronic arsenic exposure is associated with multiple adverse health effects, including cancer [World Health Organization (WHO) 2001]. Arsenic easily crosses the placenta (Concha et al. 1998), and there is increasing evidence that even moderate exposure during pregnancy is associated with fetal loss (Rahman et al. 2010), impaired fetal growth (Hopenhayn et al. 2003; Rahman et al. 2009; Yang et al. 2003), and increased infant morbidity (Rahman et al. 2011). Several studies conducted in Mexico (Rosado et al. 2007), China (Wang et al. 2007), India (von Ehrenstein et al. 2007), and Bangladesh (Hamadani et al. 2011; Wasserman et al. 2007) indicated associations between drinking-water arsenic exposure and impaired cognitive development in school-age children.

There is much less information on effects of arsenic on child growth. Two cross-sectional studies in Bangladesh reported associations of arsenic in drinking water with low body mass index (BMI) (Watanabe et al. 2007) and increased wasting (Minamoto et al. 2005) in schoolchildren. Similarly, a cross-sectional study in China suggested that elevated water arsenic concentrations were inversely associated with growth of children 8–12 years of age (Wang et al. 2007). However, there are no data from longitudinal studies.

In our ongoing mother–child cohort in rural Bangladesh, we observed that arsenic exposure during pregnancy was associated with lower birth weight (Rahman et al. 2009). In the present study, we tested the hypothesis that arsenic exposure continues to affect weight and length during infancy and early childhood.

## Materials and Methods

*Study area and population.* This study was conducted in Matlab, a rural area 53 km southeast of Dhaka. Matlab is the field site of the International Centre for Diarrhoeal Disease Research, Bangladesh (ICDDR,B) where a Health and Demographic Surveillance System (HDSS) has been in place for collecting vital information since 1966 (ICDDR,B 2006). Farming is the main source of income, followed by fishing and trading (Razzaque et al. 2007). More than 95% of the population uses tube wells for drinking water (ICDDR,B 1998). Our parallel study (Rahman et al. 2006) showed that approximately 70% of the 13,286 tube wells had arsenic concentrations above the WHO guideline of 10 µg/L (WHO 2006a). Water consumed by the present cohort of pregnant women had a median (10th–90th percentiles) arsenic concentration of 78 µg/L (1–410 µg/L) (Vahter et al. 2006).

*Study design.* This study was nested in a large community-based, randomized trial (MINIMat; Maternal and Infant Nutrition Interventions, Matlab), designed to investigate the effects of antenatal food and micronutrient supplementations on pregnancy outcomes and child health and development (Saha et al. 2009; Tofail et al. 2008). Women were enrolled from November 2001 through October 2003 after testing positive in pregnancy test at about 8 weeks of gestation. Weight and length were measured at birth and until 2 years of age. Data on household socioeconomic status (SES) and maternal weight and height were collected at enrollment.

All infants born before the end of December 2003 (*n* = 2,853; first birth, May 2002) with arsenic measurements at 18 months of age (*n* = 2,372) were selected for this study. Arsenic exposure was estimated based on concentrations of arsenic metabolites in urine (U-As), which provide a measure of inorganic arsenic exposure from all sources. U-As measurements were available for 2,096 and 2,170 of the mothers at 8 and 30 weeks of pregnancy, respectively. The main reason for missing U-As data was a delay in initiation of urine sampling in early pregnancy.

*Measurement of urinary arsenic.* We collected urine samples from women with positive results in the pregnancy test, usually at about 8 weeks gestation, and again at their clinic visit at 30 weeks gestation (Vahter et al. 2006). Urine was also collected from the children at their homes at 18 months of age (Fangstrom et al. 2009). The urine samples were transported to the ICDDR,B hospital laboratory within 6 hr and stored at –70°C. They were then transported frozen to Karolinska Institutet, Stockholm, where they were kept at –80°C until analysis.

Maternal urine samples were analyzed for metabolites of inorganic arsenic (sum of methylarsonic acid, dimethylarsinic acid, and remaining unmethylated inorganic arsenic) using hydride generation atomic absorption spectroscopy method (Vahter et al. 2006). Children’s urine samples were analyzed for the different arsenic metabolites using high pressure liquid chromatography online with hydride generation and inductively coupled plasma mass spectrometry (Fangstrom et al. 2009). We applied adequate quality control, showing excellent agreement between the two methods (Spearman *r* = 0.98; *n* = 319) as well as with reference materials (Fangstrom et al. 2008; Vahter et al. 2006). Arsenic concentrations were adjusted for variations in urine dilution by specific gravity (mean, 1.012 g/mL for maternal urine, 1.009 g/mL for child urine) (Nermell et al. 2008).

*Anthropometry.* Weight and length were measured at birth, every month in the first year of life and quarterly in the second (Saha et al. 2009), using electronic or beam scales that were precise to 10 g (UNICEF Uniscale; SECA Gmbh & Co, Hamburg, Germany). Locally manufactured, collapsible length boards that were precise to 1 mm were used to measure recumbent length. Maternal weights were measured using electronic scales (Uniscale; SECA) that were precise to 100 g. Interviewers who collected birth measurements and infant follow-up data were specially trained on anthropometric measurements.

Weight and length measurements were converted to *z*-scores – weight for age (WAZ) and length for age (LAZ), according to the WHO Multicenter Growth Reference Study child growth standards (WHO 2006b).Underweight was defined as WAZ < –2 and stunting as LAZ < –2. Maternal BMI (kilograms per meter squared) was calculated from maternal weight and height at enrollment.

*SES.* We used a wealth index as a measure of SES, which was created based on information on household assets, including land, construction materials of the houses, household assets, number of certain clothes and shoes owned (Saha et al. 2008a; Tofail et al. 2008). Principal component analysis was used to create this index, as described in detail elsewhere (Saha et al. 2008b). Households were classified into SES quintiles.

*Statistical methods.* We first evaluated the associations between U-As and anthropometric measures using scatterplots and comparison of mean weight and length in children at 3–24 months of age across quintiles of maternal and child U-As concentrations. Testing for linear trends was based on median U-As in each quintile. We then used general linear regression models to examine associations between quintiles of maternal or child U-As (with lowest quintile as reference group) and the attained weight or length of infants and young children (adjusting for the small variation in age within each age group). The models were then adjusted for child sex and maternal BMI and SES, which were found to influence the associations of U-As with both weight and length. Residual analysis indicated no major deviation from a linear pattern. As sensitivity analyses, we additionally adjusted for birth weight or length, and maternal U-As. We repeated the analyses stratified by child sex and SES (median split).

Because we previously found that underweight and stunting were prevalent in this cohort (Saha et al. 2009), we evaluated potential associations of U-As (quintiles) with these measures using logistic regression analysis. Goodness of fit was checked with the Hosmer–Lemeshow test (> 0.05).

Statistical analyses were carried out using SPSS version 18.0 (SPSS, Inc., Chicago, IL, USA). All tests were two-sided, and *p* < 0.05 was considered statistically significant.

*Ethical considerations.* Mothers were informed about the study and their written consent was obtained at the time of enrollment during pregnancy. For 18-month tests on the children, written consent was obtained from parents or guardians after briefing them about the study. The project was approved by the ethical committees at ICDDR,B and Karolinska Institutet. Our parallel screening project for water arsenic (Rahman et al. 2006) included a mitigation component, in which tube wells exceeding the local drinking water standard for arsenic were painted red to indicate that they should not be used. People were encouraged to use wells with low arsenic concentrations (painted green), and were given assistance to find other means to decrease arsenic exposure (Jakariya et al. 2005).

## Results

At 3 months of age, 66% of the children had data on anthropometry, but data completeness increased as the children got older, reaching 94% at 12 months and during the second year of life. We had low coverage of anthropometry measurements in the first year but, with time, more field workers were employed and trained and the coverage improved. Eighty-eight percent and 91% of the children had information on their mothers’ U-As at 8 and 30 weeks gestation, respectively.

We compared children with and without missing anthropometry data according to baseline characteristics (birth weight, birth length, maternal U-As in early and late pregnancy, child U-As, and SES) and found no significant differences between groups (data not shown). Similarly, we compared children of mothers who did (*n* = 2,087 at 8 weeks gestation; *n* = 2,159 at 30 weeks gestation) or did not (*n* = 285 at 8 weeks gestation; *n* = 213 at 30 weeks gestation) provide urine samples for arsenic measurements, and found no significant differences between groups (data not shown).

Infants were small at birth (Table 1); about 31% of infants were born with low birth weight (< 2,500 g). They continued to be small for age during the first 2 years of life (Tables 2 and 3), at which time 40.4% and 52.5% of the boys and 37.1% and 48.8% of the girls were underweight and stunted, respectively. About 30% of the mothers had a BMI < 18.5 kg/m^2^ at enrollment (~ 8 weeks gestation), which indicates that they may have been undernourished. Median maternal U-As in pregnancy was about 80 µg/L, and median child U-As at 18 months of age was 34 µg/L.

**Table 1:** General and baseline characteristics of the study cohort.

Variable	n	Mean ± SD	Median (10th–90th percentiles)
Boys (%)		1,239		52		
Girls (%)		1,133		48		
Birth weight (g)		2,246		2,698 ± 392		2,680 (2,223–3,205)
Boys				2,733 ± 409		2,720 (2,243–3,271)
Girls				2,659 ± 369		2,642 (2,200–3,140)
Birth length (cm)		2,240		47.8 ± 2.1		48.0 (45.1–50.3)
Boys				48.0 ± 2.2		48.0 (45.4–50.7)
Girls				47.5 ± 2.0		47.6 (45.0–50.0)
Mother’s age at enrollment (years)		2,372		27.0 ± 5.9		26.5 (19.6–35.0)
Mother’s BMI (kg/m2)a		2,363		20.1 ± 2.6		19.7 (17.2–23.8)
Mother’s U-As at 8 weeks gestation (µg/L)		2,096		153 ± 181		80.9 (23.4–378)
Mother’s U-As at 30 weeks gestation (µg/L)		2,170		167 ± 196		84.0 (24.8–414)
Child’s U-As at 18 months (µg/L)		2,372		66.8 ± 87.7		34.2 (11.7–159)
U-As in boys				64.9 ± 85.1		33.6 (11.5–153)
U-As in girls				68.8 ± 90.5		35.8 (11.9–170)
aMeasured at enrollment.						

**Table 2:** Attained child weight (kg) at 3, 6, 9, 12, 18, and 24 months of age (mean ± SD) by quintiles (Q) of maternal U-As (µg/L) at 8 and 30 weeks gestation.

Quintile of maternal U-As [median (range)]	3 months	6 months	9 months	12 months	18 months	24 months
Overall mean weight		5.29 ± 0.72		6.73 ± 0.89		7.46 ± 1.01		7.99 ± 1.08		8.87 ± 1.14		9.72 ± 1.21
At 8 weeks gestation												
Q1: 23 (1.2–33)		5.29 ± 0.72		6.82 ± 0.89		7.54 ± 1.06		8.12 ± 1.16		9.02 ± 1.27		9.85 ± 1.32
Q2: 41 (33–57)		5.37 ± 0.71		6.82 ± 0.88		7.54 ± 1.00		8.04 ± 1.15		8.91 ± 1.19		9.77 ± 1.27
Q3: 80 (57–115)		5.21 ± 0.73		6.65 ± 0.89		7.39 ± 1.00		7.91 ± 1.02		8.80 ± 1.03		9.64 ± 1.14
Q4: 169 (116–245)		5.33 ± 0.69		6.68 ± 0.91		7.40 ± 1.00		7.90 ± 1.03		8.80 ± 1.12		9.65 ± 1.16
Q5: 378 (246–1,611)		5.27 ± 0.71		6.69 ± 0.88		7.42 ± 1.00		7.98 ± 1.03		8.84 ± 1.09		9.69 ± 1.15
p-Value for trenda		0.47		0.067		0.10		0.21		0.13		0.19
At 30 weeks gestation												
Q1: 25 (1.8–36)		5.38 ± 0.72		6.79 ± 0.93		7.53 ± 1.04		8.06 ± 1.15		8.96 ± 1.32		9.82 ± 1.42
Q2: 48 (36–63)		5.33 ± 0.70		6.79 ± 0.89		7.50 ± 1.06		8.03 ± 1.08		8.88 ± 1.14		9.75 ± 1.20
Q3: 84 (63–120)		5.30 ± 0.72		6.75 ± 0.89		7.45 ± 0.97		7.96 ± 1.10		8.89 ± 1.13		9.70 ± 1.18
Q4: 185 (121–272)		5.27 ± 0.69		6.74 ± 0.89		7.45 ± 0.99		7.94 ± 1.05		8.86 ± 1.08		9.66 ± 1.14
Q5: 414 (273–1,632)		5.21 ± 0.73		6.66 ± 0.89		7.35 ± 0.97		7.90 ± 1.01		8.85 ± 1.08		9.63 ± 1.12
p-Value for trenda		0.003		0.026		0.012		0.038		0.31		0.042
aLinear trends across categories were tested using the median U-As concentrations within categories as continuous variable.												

**Table 3:** Attained child length (cm) at 3, 6, 9, 12, 18, and 24 months of age (mean ± SD) by quintiles (Q) of maternal U-As (µg/L) at 8 and 30 weeks gestation.

Quintile of maternal U-As [median (range)]	3 months	6 months	9 months	12 months	18 months	24 months
Overall mean length		57.8 ± 2.3		63.6 ± 2.5		67.7 ± 2.5		70.8 ± 2.7		75.9 ± 3.9		80.5 ± 3.4
At 8 weeks gestation												
Q1: 23 (1.2–33)		57.9 ± 2.4		63.9 ± 2.5		67.9 ± 2.7		71.1 ± 2.8		76.2 ± 3.3		80.8 ± 3.5
Q2: 41 (33–57)		58.0 ± 2.3		63.7 ± 2.4		67.6 ± 2.4		70.8 ± 2.6		75.8 ± 2.9		80.6 ± 3.1
Q3: 80 (57–115)		57.6 ± 2.2		63.5 ± 2.5		67.5 ± 2.6		70.7 ± 2.7		75.7 ± 3.0		80.3 ± 3.4
Q4: 169 (116–245)		57.8 ± 2.3		63.4 ± 2.6		67.6 ± 2.7		70.6 ± 2.8		75.8 ± 3.3		80.2 ± 3.5
Q5: 378 (246–1,611)		57.9 ± 2.3		63.7 ± 2.3		67.7 ± 2.4		70.7 ± 2.6		76.0 ± 3.0		80.6 ± 3.2
p-Value for trenda		0.89		0.60		0.88		0.095		0.94		0.76
At 30 weeks gestation												
Q1: 25 (1.8–36)		58.2 ± 2.3		63.9 ± 2.5		67.8 ± 2.5		71.1 ± 2.7		76.1 ± 3.3		80.9 ± 3.5
Q2: 48 (36–63)		58.0 ± 2.3		63.7 ± 2.5		67.7 ± 2.7		70.8 ± 2.7		75.9 ± 3.2		80.5 ± 3.3
Q3: 84 (63–120)		57.8 ± 2.3		63.7 ± 2.4		67.7 ± 2.5		70.8 ± 2.7		75.9 ± 3.0		80.5 ± 3.4
Q4: 185 (121–272)		57.7 ± 2.2		63.6 ± 2.3		67.8 ± 2.5		70.8 ± 2.6		75.9 ± 3.0		80.5 ± 3.3
Q5: 414 (273–1,632)		57.6 ± 2.3		63.4 ± 2.6		67.5 ± 2.6		70.6 ± 2.7		75.8 ± 3.0		80.4 ± 3.2
p-Value for trenda		0.009		0.003		0.070		0.017		0.19		0.11
aLinear trends across categories were tested using the median U-As concentrations within categories as continuous variable.												

*Association of prenatal arsenic exposure with attained weight and length.* Mean attained weight of the children generally decreased with increasing quintiles of maternal U-As, particularly at 30 weeks gestation (Table 2). With the latter exposure there was a significant linear trend of decreasing weight at all time points except 18 months. When stratified by sex, this pattern appeared to be limited to girls, without linear trends in boys [see Supplemental Material, Table 1 (http://dx.doi.org/10.1289/ehp.103378)]. Overall results as well as results stratified by sex followed a similar pattern for children’s length (Table 3; see Supplemental Material, Table 2).

In the unadjusted linear regression analysis, quintiles of maternal U-As at both 8 and 30 weeks gestation (with first quintile as reference) were inversely associated with attained child weight (Table 4). When the models were adjusted for child sex and maternal BMI and SES, the associations were generally close to the null. Results from stratified analysis by child sex were similar [see Supplemental Material Table 3 (http://dx.doi.org/10.1289/ehp.103378)]. Additional adjustment by birth weight further changed the estimates (on average ~ 50%) (data not shown). Corresponding analyses with child length produced similar results (Table 5). Most of the adjusted estimates for both weight and length among boys were positive in the highest quintile, whereas those for girls were negative (Supplemental Material Tables 3 and 4).

**Table 4:** Multiple-adjusted regression analysis for evaluation of associations between quintiles (Q) of maternal U-As (µg/L) at 8 and 30 weeks gestation and attained weight (kg) of children at 3, 6, 9, 12, 18, and 24 months of age [β (95% confidence interval)].

Quintile of maternal U-As [median (range)]	3 months	6 months	9 months	12 months	18 months	24 months
At 8 weeks gestation											
Unadjusteda											
Q1: 23 (1.2–33)	Reference		Reference		Reference		Reference		Reference		Reference
Q2: 41 (33–57)	0.080 (–0.033, 0.19)		0.022 (–0.11, 0.15)		–0.012 (–0.16, 0.14)		–0.078 (–0.23, 0.073)		–0.11 (–0.27, 0.056)		–0.073 (–0.25, 0.10)
Q3: 80 (57–115)	–0.080 (–0.19, 0.034)		–0.17 (–0.30, –0.035)		–0.16 (–0.30, –0.008)		–0.21 (–0.36, –0.063)		–0.23 (–0.39, –0.073)		–0.20 (–0.38, –0.033)
Q4: 169 (116–245)	0.032 (–0.087, 0.15)		–0.12 (–0.25, 0.018)		–0.16 (–0.30, –0.009)		–0.22 (–0.37, –0.070)		–0.22 (–0.38, –0.059)		–0.20 (–0.37, –0.026)
Q5: 378 (246–1,611)	–0.020 (–0.14, 0.096)		–0.12 (–0.26, 0.010)		–0.13 (–0.27, 0.019)		–0.14 (–0.29, 0.015)		–0.18 (–0.34, –0.015)		–0.15 (–0.33, 0.020)
Adjustedb											
Q1: 23 (1.2–33)	Reference		Reference		Reference		Reference		Reference		Reference
Q2: 41 (33–57)	0.11 (0.006, 0.21)		0.070 (–0.050, 0.19)		0.044 (–0.087, 0.18)		–0.022 (–0.16, 0.11)		–0.033 (–0.18, 0.11)		–0.015 (–0.17, 0.14)
Q3: 80 (57–115)	–0.017 (–0.12, 0.086)		–0.079 (–0.20, 0.04)		–0.060 (–0.19, 0.071)		–0.13 (–0.26, 0.005)		–0.12 (–0.26, 0.024)		–0.092 (–0.25, 0.062)
Q4: 169 (116–245)	0.089 (–0.019, 0.20)		–0.027 (–0.15, 0.095)		–0.042 (–0.17, 0.089)		–0.090 (–0.22, 0.045)		–0.065 (–0.21, 0.079)		–0.060 (–0.22, 0.094)
Q5: 378 (246–1,611)	0.078 (–0.028, 0.18)		0.015 (–0.11, 0.14)		0.019 (–0.11, 0.15)		0.027 (–0.11, 0.16)		0.024 (–0.12, 0.17)		0.044 (–0.11, 0.20)
At 30 weeks gestation											
Unadjusteda											
Q1: 25 (1.8–36)	Reference		Reference		Reference		Reference		Reference		Reference
Q2: 48 (36–63)	–0.047 (–0.16, 0.068)		0.005 (–0.13, 0.14)		–0.037 (–0.18, 0.11)		–0.023 (–0.17, 0.13)		–0.092 (–0.25, 0.067)		–0.066 (–0.24, 0.10)
Q3: 84 (63–120)	–0.089 (–0.20, 0.027)		–0.028 (–0.16, 0.11)		–0.087 (–0.23, 0.060)		–0.094 (–0.24, 0.054)		–0.078 (–0.24, 0.081)		–0.12 (–0.29, 0.050)
Q4:185 (121–272)	–0.12 (–0.24, 0.002)		–0.046 (–0.18, 0.089)		–0.084 (–0.23, 0.061)		–0.11 (–0.26, 0.038)		–0.096 (–0.26, 0.064)		–0.16 (–0.33, 0.013)
Q5: 414 (273–1,632)	–0.18 (–0.29, –0.058)		–0.13 (–0.27, 0.006)		–0.19 (–0.33, –0.040)		–0.15 (–0.30, –0.002)		–0.11 (–0.27, 0.048)		–0.19 (–0.36, –0.016)
Adjustedb											
Q1: 25 (1.8–36)	Reference		Reference		Reference		Reference		Reference		Reference
Q2: 48 (36–63)	–0.002 (–0.11, 0.10)		0.059 (–0.063, 0.18)		0.018 (–0.11, 0.15)		0.020 (–0.11, 0.15)		–0.015 (–0.16, 0.13)		0.0004 (–0.15, 0.15)
Q3: 84 (63–120)	–0.063 (–0.17, 0.042)		–0.011 (–0.13, 0.11)		–0.097 (–0.23, 0.033)		–0.11 (–0.24, 0.024)		–0.066 (–0.21, 0.076)		–0.11 (–0.26, 0.044)
Q4:185 (121–272)	–0.066 (–0.17, 0.042)		–0.002 (–0.13, 0.12)		–0.026 (–0.15, 0.10)		–0.064 (–0.20, 0.067)		–0.017 (–0.16, 0.13)		–0.083 (–0.24, 0.070)
Q5: 414 (273–1,632)	–0.073 (–0.18, 0.035)		0.004 (–0.12, 0.13)		–0.064 (–0.20, 0.066)		–0.023 (–0.16, 0.11)		0.047 (–0.097, 0.19)		–0.017 (–0.17, 0.14)
aU-As (quintiles of U-As at 8 and 30 weeks gestation) and actual age in respective age group were entered. bU-As, age, sex, and maternal BMI (continuous variable) and SES (quintiles as continuous variable) were entered.											

**Table 5:** Multiple-adjusted linear regression analysis for evaluation of associations between quintiles (Q) of maternal U-As (µg/L) at 8 and 30 weeks gestation and attained length (cm) of children at 3, 6, 9, 12, 18, and 24 months of age [β (95% confidence interval)].

Quintile of maternal U-As [median (range)]	3 months	6 months	9 months	12 months	18 months	24 months
At 8 weeks gestation											
Unadjusteda											
Q1: 23 (1.2–33)	Reference		Reference		Reference		Reference		Reference		Reference
Q2: 41 (33–57)	0.15 (–0.22, 0.51)		–0.14 (–0.50, 0.23)		–0.23 (–0.61, 0.14)		–0.32 (–0.70, 0.057)		–0.42 (–0.85, 0.014)		–0.24 (–0.72, 0.24)
Q3: 80 (57–115)	–0.21 (–0.57, 0.16)		–0.38 (–0.75, –0.19)		–0.16 (–0.76,–0.015)		–0.40 (–0.78, –0.024)		–0.53 (–0.96, –0.092)		–0.52 (–0.99, –0.041)
Q4: 169 (116–245)	–0.038 (–0.42, 0.35)		–0.42 (–0.79, –0.046)		–0.16 (–0.69, 0.061)		–0.54 (–0.92, –0.16)		–0.46 (–0.90, –0.029)		–0.55 (–1.03, –0.082)
Q5: 378 (246–1,611)	0.014 (–0.36, 0.39)		–0.17 (–0.54, 0.20)		–0.13 (–0.54, 0.020)		–0.43 (–0.80, –0.052)		–0.24 (–0.67, 0.20)		–0.22 (–0.69, 0.26)
Adjustedb											
Q1: 23 (1.2–33)	Reference		Reference		Reference		Reference		Reference		Reference
Q2: 41 (33–57)	0.21 (–0.13, 0.56)		–0.055 (–0.39, 0.28)		–0.11 (–0.45, 0.22)		–0.22 (–0.56, 0.12)		–0.26 (–0.65, 0.13)		–0.098 (–0.53, 0.34)
Q3: 80 (57–115)	–0.067 (–0.41, 0.28)		–0.19 (–0.52, 0.14)		–0.20 (–0.53, 0.14)		–0.21 (–0.55, 0.12)		–0.25 (–0.64, 0.14)		–0.22 (–0.65, 0.22)
Q4: 169 (116–245)	0.094 (–0.27, 0.45)		–0.21 (–0.55, 0.13)		–0.059 (–0.39, 0.28)		–0.23 (–0.57, 0.11)		–0.060 (–0.45, 0.33)		–0.15 (–0.59, 0.28)
Q5: 378 (246–1,611)	0.26 (–0.096, 0.61)		0.14 (–0.19, 0.48)		0.13 (–0.21, 0.46)		–0.089 (–0.43, 0.25)		0.25 (–0.14, 0.65)		0.29 (–0.15, 0.73)
At 30 weeks gestation											
Unadjusteda											
Q1: 25 (1.8–36)	Reference		Reference		Reference		Reference		Reference		Reference
Q2: 48 (36–63)	–0.23 (–0.59, 0.14)		–0.20 (–0.57, 0.17)		–0.12 (–0.49, 0.25)		–0.29 (–0.66, 0.082)		–0.25 (–0.68, –0.18)		–0.39 (–0.61, 0.077)
Q3: 84 (63–120)	–0.47 (–0.84, –0.10)		–0.14 (–0.51, 0.23)		–0.10 (–0.47, 0.27)		–0.32 (–0.69, –0.27)		–0.16 (–0.59, 0.27)		–0.40 (–0.75, 0.070)
Q4: 185 (121–272)	–0.49 (–0.87, –0.12)		–0.24 (–0.61, 0.13)		–0.063 (–0.43, 0.30)		–0.28 (–0.65, 0.27)		–0.16 (–0.59, 0.27)		–0.43 (–0.63, 0.036)
Q5: 414 (273–1,632)	–0.59 (–0.96, –0.21)		–0.55 (–0.93, –0.18)		–0.35 (–0.72, 0.027)		–0.54 (–0.91, 0.082)		–0.35 (–0.78, 0.082)		–0.53 (–1.00, –0.061)
Adjustedb											
Q1: 25 (1.8–36)	Reference		Reference		Reference		Reference		Reference		Reference
Q2: 48 (36–63)	–0.11 (–0.45, 0.24)		–0.038 (–0.37, 0.30)		0.019 (–0.31, 0.35)		–0.17 (–0.50, 0.16)		–0.031 (–0.42, 0.36)		–0.18 (–0.61, 0.25)
Q3: 84 (63–120)	–0.41 (–0.76, –0.060)		–0.099 (–0.43, 0.24)		–0.12 (–0.45, 0.21)		–0.35 (–0.68, –0.014)		–0.011 (–0.50, 0.28)		–0.32 (–0.75, 0.11)
Q4: 185 (121–272)	–0.37 (–0.73, –0.017)		–0.14 (–0.47, 0.20)		0.061 (–0.27, 0.39)		–0.17 (–0.50, 0.16)		0.043 (–0.35, 0.43)		–0.21 (–0.63, 0.22)
Q5: 414 (273–1,632)	–0.35 (–0.70, 0.011)		–0.24 (–0.58, 0.10)		–0.091 (–0.43, 0.24)		–0.26 (–0.59, 0.076)		0.046 (–0.35, 0.44)		–0.087 (–0.52, 0.34)
aU-As (quintiles of U-As at 8 and 30 weeks gestation) and actual age in respective age group were entered. bU-As, age, sex, and maternal BMI (continuous variable) and SES (quintiles as continuous variable) were entered.											

*Association of postnatal arsenic exposure with subsequent attained weight and length.* Mean body weight at all ages studied (18, 21, and 24 months) decreased about 300 g from the first to the fourth quintile of child U-As at 18 months [see Supplemental Table 5 (http://dx.doi.org/10.1289/ehp.103378)]. In general, mean weight increased again at the highest quintile. When stratified by sex the overall pattern was observed in girls, but there was little evidence of a trend in boys. Similarly, child length decreased by about 1 cm from first to fourth quintile at all ages, but only in girls (see Supplemental Material, Table 6). Here too, mean length increased again at highest quintile.

**Table 6:** Multiple-adjusted linear regression analysis for evaluation of associations between quintiles (Q) of child U-As (µg/L) at 18 months of age and attained weight (kg) at 18, 21, and 24 months of age [β (95% confidence interval)].

Quintile of U-As at 18 months [median (range)]	18 months	21 months	24 months
All children			
Unadjusteda			
Q1: 12 (2.4–16)	Reference	Reference	Reference
Q2: 20 (16–26)	–0.24 (–0.39, –0.091)	–0.15 (–0.31, 0.004)	–0.19 (–0.35, –0.022)
Q3: 34 (26–46)	–0.29 (–0.44, –0.14)	–0.24 (–0.40, –0.084)	–0.25 (–0.42, –0.090)
Q4: 64 (46–96)	–0.36 (–0.51, –0.21)	–0.29 (–0.45, –0.14)	–0.28 (–0.44, –0.11)
Q5: 159 (96–937)	–0.22 (–0.37, –0.068)	–0.23 (–0.38, –0.068)	–0.17 (–0.33, –0.004)
Adjustedb			
Q1: 12 (2.4–16)	Reference	Reference	Reference
Q2: 20 (16–26)	–0.097 (–0.23, 0.038)	–0.013 (–0.15, 0.13)	–0.027 (–0.18, 0.12)
Q3: 34 (26–46)	–0.18 (–0.32, –0.047)	–0.14 (–0.28, –0.001)	–0.13 (–0.28, 0.019)
Q4: 64 (46–96)	–0.19 (–0.33,–0.57)	–0.13 (–0.27, 0.011)	–0.082 (–0.23, 0.067)
Q5: 159 (96–937)	–0.059 (–0.20, 0.076)	–0.073 (–0.22, 0.069)	0.005 (–0.14, 0.16)
Boys			
Unadjusteda			
Q1: 12 (2.4–16)	Reference	Reference	Reference
Q2: 20 (16–26)	–0.18 (–0.39, 0.029)	–0.059 (–0.27, 0.16)	–0.11 (–0.34, 0.12)
Q3: 34 (26–46)	–0.14 (–0.34, 0.066)	–0.13 (–0.34, 0.082)	–0.10 (–0.33, 0.12)
Q4: 64 (46–96)	–0.21 (–0.42, –0.002)	–0.15 (–0.37, 0.061)	–0.13 (–0.36, 0.10)
Q5: 159 (96–937)	–0.081 (–0.29, 0.13)	–0.044 (–0.26, 0.18)	–0.011 (–0.24, 0.22)
Adjustedc			
Q1: 12 (2.4–16)	Reference	Reference	Reference
Q2: 20 (16–26)	–0.043 (–0.24, 0.15)	0.06 (–0.14, 0.26)	0.041 (–0.17, 0.26)
Q3: 34 (26–46)	0.012 (–0.18, 0.20)	0.015 (–0.18, 0.21)	0.057 (–0.15, 0.27)
Q4: 64 (46–96)	–0.060 (–0.26, 0.14)	–0.003 (–0.20, 0.20)	0.038 (–0.18, 0.26)
Q5: 159 (96–937)	0.028 (–0.17, 0.22)	0.051 (–0.15, 0.26)	0.12 (–0.096, 0.34)
Girls			
Unadjusteda			
Q1: 12 (2.4–16)	Reference	Reference	Reference
Q2: 20 (16–26)	–0.28 (–0.48, –0.081)	–0.22 (–0.43, –0.010)	–0.22 (–0.44,–0.008)
Q3: 34 (26–46)	–0.50 (–0.71, –0.30)	–0.39 (–0.60, –0.18)	–0.46 (–0.68, –0.24)
Q4: 64 (46–96)	–0.48 (–0.68, –0.28)	–0.41 (–0.62, –0.20)	–0.37 (–0.58, –0.15)
Q5: 159 (96–937)	–0.33 (–0.52, –0.13)	–0.36 (–0.57, –0.15)	–0.27 (–0.49, –0.055)
Adjustedc			
Q1: 12 (2.4–16)	Reference	Reference	Reference
Q2: 20 (16–26)	–0.16 (–0.35, 0.026)	–0.10 (–0.30, 0.093)	–0.11 (–0.31, 0.094)
Q3: 34 (26–46)	–0.41 (–0.60, –0.22)	–0.32 (–0.52, –0.12)	–0.35 (–0.56, –0.14)
Q4: 64 (46–96)	–0.34 (–0.52, –0.15)	–0.27 (–0.47, –0.075)	–0.22 (–0.42, –0.014)
Q5: 159 (96–937)	–0.16 (–0.35, 0.023)	–0.22 (–0.42, –0.022)	–0.13 (–0.34, 0.074)
aU-As (quintiles of U-As at 18 months) and actual age in respective age group were entered. bU-As, age, sex and maternal BMI (continuous variable) and SES (quintiles as continuous variable) were entered. cU-As, age, and maternal BMI (continuous variable) and SES (quintiles as continuous variable) were entered.			

In the unadjusted regression analysis, essentially all quintiles of U-As at 18 months were significantly inversely associated with concurrent and subsequent attained weights, but these associations decreased markedly after adjusting for child sex and maternal BMI and SES (Table 6). In the sex-stratified analyses, the inverse associations of U-As with child weight were present only among the girls. Although adjustment decreased the estimates by 20–50%, associations were still statistically significant at the third and fourth quintiles of U-As in the girls. Associations were weaker in the highest quintile compared with the fourth quintile. Additional adjustment by maternal U-As (30 weeks gestation) did not change the estimates, and adjustment by birth weight only slightly decreased them (~ 15%) (data not shown). Stratification by SES showed stronger inverse associations among girls in higher SES groups (above median) than in lower (data not shown). Corresponding analyses for U-As associations with attained length gave similar results (Table 7). Inverse associations were found only among the girls, and adjusted associations were statistically significant in the third and fourth quintiles of U-As. Estimates decreased by 10–50% after additional adjustment for birth length but not by maternal U-As (data not shown).

**Table 7:** Multiple-adjusted linear regression analysis for evaluation of associations between quintiles (Q) of child U-As (µg/L) at 18 months of age and attained length (cm) at 18, 21, and 24 months of age [β (95% confidence interval)].

Quintile of U-As at 18 months [median (range)]	18 months	21 months	24 months
All children			
Unadjusteda			
Q1: 12 (2.4–16)	Reference	Reference	Reference
Q2: 20 (16–26)	–0.22 (–0.62, 0.19)	–0.37 (–0.80, 0.055)	–0.27 (–0.71, 0.17)
Q3: 34 (26–46)	–0.55 (–0.96, –0.14)	–0.52 (–0.95, –0.089)	–0.52 (–0.97, –0.075)
Q4: 64 (46–96)	–0.47 (–0.88, –0.067)	–0.73 (–1.16, –0.030)	–0.80 (–1.24, –0.35)
Q5: 159 (96–937)	–0.23 (–0.64, 0.18)	–0.45 (–0.88, –0.014)	–0.47 (–0.91, –0.019)
Adjustedb			
Q1: 12 (2.4–16)	Reference	Reference	Reference
Q2: 20 (16–26)	0.16 (–0.21, 0.53)	–0.003 (–0.40, 0.39)	0.16 (–0.25, 0.57)
Q3: 34 (26–46)	–0.28 (–0.65, 0.093)	–0.26 (–0.66, 0.13)	–0.18 (–0.59, 0.23)
Q4: 64 (46–96)	–0.024 (–0.39, 0.35)	–0.28 (–0.67, 0.12)	–0.26 (–0.67, 0.15)
Q5: 159 (96–937)	0.18 (–0.20, 0.55)	–0.070 (–0.47, 0.33)	–0.013 (–0.43, 0.40)
Boys			
Unadjusteda			
Q1: 12 (2.4–16)	Reference	Reference	Reference
Q2: 20 (16–26)	0.14 (–0.41, 0.69)	–0.12 (–0.70, 0.47)	–0.094 (–0.71, 0.52)
Q3: 34 (26–46)	–0.22 (–0.77, 0.32)	–0.29 (–0.87, 0.28)	–0.17 (–0.77, 0.44)
Q4: 64 (46–96)	–0.12 (–0.67, 0.44)	–0.36 (–0.95, 0.22)	–0.40 (–1.02, 0.22)
Q5: 159 (96–937)	0.23 (–0.33, 0.79)	–0.002 (–0.60, 0.60)	–0.018 (–0.64, 0.61)
Adjustedc			
Q1: 12 (2.4–16)	Reference	Reference	Reference
Q2: 20 (16–26)	0.49 (–0.039, 1.01)	0.18 (–0.38, 0.73)	0.29 (–0.29, 0.87)
Q3: 34 (26–46)	0.13 (–0.38, 0.64)	0.067 (–0.48, 0.61)	0.26 (–0.31, 0.83)
Q4: 64 (46–96)	0.29 (–0.23, 0.82)	0.049 (–0.50, 0.60)	0.072 (–0.52, 0.66)
Q5: 159 (96–937)	0.47 (–0.052, 1.00)	0.20 (–0.36, 0.76)	0.31 (–0.28, 0.90)
Girls			
Unadjusteda			
Q1: 12 (2.4–16)	Reference	Reference	Reference
Q2: 20 (16–26)	–0.52 (–1.07, 0.031)	–0.56 (–1.14, 0.024)	–0.36 (–0.96, 0.25)
Q3: 34 (26–46)	–0.99 (–1.56, –0.43)	–0.82 (–1.42, –0.22)	–0.99 (–1.62, –0.37)
Q4: 64 (46–96)	–0.77 (–1.32, –0.22)	–1.02 (–1.61, –0.44)	–1.07 (–1.67, –0.47)
Q5: 159 (96–937)	–0.62 (–1.17, –0.065)	–0.76 (–1.35, –0.17)	–0.78 (–1.39, –0.18)
Adjustedc			
Q1: 12 (2.4–16)	Reference	Reference	Reference
Q2: 20 (16–26)	–0.20 (–0.72, 0.32)	–0.23 (–0.79, 0.32)	–0.025 (–0.60, 0.55)
Q3: 34 (26–46)	–0.74 (–1.27, –0.21)	–0.65 (–1.22, –0.080)	–0.71 (–1.30, –0.12)
Q4: 64 (46–96)	–0.38 (–0.90, 0.14)	–0.65 (–1.21, –0.088)	–0.64 (–1.21, –0.058)
Q5: 159 (96–937)	–0.17 (–0.70, 0.35)	–0.40 (–0.69, 0.16)	–0.39 (–0.97, 0.19)
aU-As (quintiles of U-As at 18 months) and actual age in respective age group were entered. bU-As, age, sex and maternal BMI (continuous variable) and SES (quintiles as continuous variable) were entered. cU-As, age, and maternal BMI (continuous variable) and SES (quintiles as continuous variable) were entered.			

**Figure 1:**
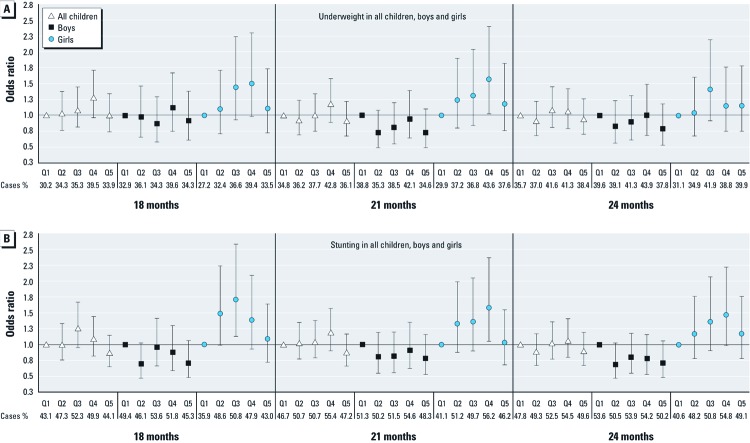
Odds ratios (95% confidence intervals) of underweight (weight for age *z*-scores < –2) (*A*) and stunting (length for age *z*-scores < –2) (*B*) in all children, boys, and girls at 18, 21, and 24 months of age, in relation to child U-As at 18 months. Data are adjusted for age, maternal BMI and SES, and sex (or stratified by sex).

The odds of underweight and stunting generally increased with increasing U-As quintiles up to the fourth quintile among girls, but not among boys [Figure 1; see also Supplemental Material, Tables 7 and 8 (http://dx.doi.org/10.1289/ehp.103378)]. Compared with girls in the lowest quintile of U-As (< 16 µg/L), those in the fourth quintile (46–96 µg/L) had an odds ratio (OR) for underweight (adjusted for age and maternal BMI and SES) of 1.50 (95% confidence interval: 0.98–2.30), 1.57 (1.02–2.40), and 1.15 (0.75–1.76) at 18, 21, and 24 months of age, respectively (see Supplemental Material, Table 7). The corresponding ORs for stunting were 1.39 (0.93–2.09), 1.58 (1.05–2.37), and 1.47 (0.98–2.22) (see Supplemental Material, Table 8). Most of the adjusted ORs among the boys (quintiles 2–5) were < 1, whereas all among the girls were > 1. Similar to the regression analyses of weight and length as continuous outcomes, the ORs for underweight and stunting were lower in the highest quintile than in the lower ones. Stratifying by SES did not show major differences in U-As associations by SES (data not shown), probably because many more children were underweight and stunting in the low SES group.

## Discussion

Our results suggest that arsenic exposure in early childhood influences the body weight and length more than the prenatal exposure and more in girls than in boys. Arsenic exposure was also associated with increased prevalence of underweight and stunting in the girls, although overall the prevalence was higher in boys than in girls. An apparent effect of arsenic was observed at low exposure levels, < 50 µg/L in urine. Considering the prevalence of such exposure levels world-wide, the findings have obvious public health relevance. To our knowledge, this is the first longitudinal study to investigate the relationships of arsenic exposure with body weight and length in early life.

We have previously reported evidence that arsenic exposure during pregnancy negatively affects size at birth (Rahman et al. 2009). Here, the lack of clear associations between prenatal arsenic exposure and children’s weight and length may be explained by the fact that 92% of the children were partially breast-fed until 1 year of age (Saha et al. 2008a), which efficiently protects them against arsenic exposure (Fangstrom et al. 2008). Once weaning began, children were exposed to arsenic through water and food, and our results suggest that this exposure had a negative effect on the weight and length of the children, particularly the girls. Compared with girls with U-As < 16 µg/L, those in the fourth quintile (46–96 µg/L) were approximately 300 g lighter and 0.7 cm shorter. This exposure was also associated with about 50% increased risk for underweight and stunting. Girls with arsenic exposure above median had 3% and 5% units higher prevalence of underweight and stunting, respectively, compared with below median exposure [see Supplemental Material, Figure 1 (http://dx.doi.org/10.1289/ehp.103378)]. Early childhood stunting is closely associated with poor cognitive and educational performance (Grantham-McGregor et al. 2007). In fact, at 5 years of age, children’s arsenic exposure in the present cohort was inversely associated with both Verbal and Full-Scale IQ (Hamadani et al. 2011), whereas arsenic was not associated with impaired child development at 7 or 18 months (Hamadani et al. 2010; Tofail et al. 2009). In line with the present findings, associations with lower IQ at 5 years were seen mainly in girls. Reasons for the observed differential effects of arsenic by sex remain to be elucidated.

The reason for generally lower associations at the highest quintile of exposure is unclear. One possible reason is the strong correlation between arsenic and iron concentrations in the drinking water (Spearman *r* = 0.61; *n* = 1,035). The water used by the children in the fifth quintile of U-As had on average 1.6 mg iron/L (90th percentile, 5 mg/L). Thus, consumption of only 1 L/day will contribute significantly to the total iron intake. Interestingly, the prevalence of iron deficiency in the early pregnancy mothers was unexpectedly low (Lindstrom et al. 2011). This may have resulted from consumption of iron-rich water, as was recently found for women in northern Bangladesh (Merrill et al. 2011). The potentially protective effect of iron against arsenic-related effects in children should be evaluated further.

Measurements of arsenic in urine were valid and reliable (Fangstrom et al. 2009). We chose to use U-As as that captures exposure from all sources, including food. In particular, rice, the main staple food, contained elevated arsenic concentrations (Gardner et al. 2011). Also, measurements of body weight and length were adequate to assess the rapidly changing growth early in life. The short intervals of follow-up (monthly in the first year and quarterly in the second year) were appropriate to model attained weight and length in the young children. Although we adjusted for major influential confounders, we cannot rule out the possibility that unmeasured confounders influenced the associations between exposure and body size of the children.

## Conclusion

We found inverse associations between postnatal arsenic exposure and weight and length at 1.5–2 years of age, particularly in girls. In Bangladesh, both exposure to arsenic through drinking water and child undernutrition are widespread. The fact that the children continue to be exposed (Gardner et al. 2011) emphasizes the urgent need for efficient mitigation and follow-up of child health.

## Supplemental Material

(274 KB) PDF
