# Supplementary material for: Pre- and Postnatal Arsenic Exposure and Body Size to 2 Years of Age: A Cohort Study in Rural Bangladesh
Source: Environ Health Perspect. 2012 Apr 13;120(8):1208–14. doi: 10.1289/ehp.1003378 (PMC3440068; doi:10.1289/ehp.1003378)
Supplement: (274 KB) PDF [file ehp.1003378.s001.pdf]

Supplemental Material

Pre- and Postnatal Arsenic Exposure and Body Size to Two Years of Age: a Cohort Study in Rural Bangladesh

Kuntal K. Saha[1, 2] Annette Engström,[3] Jena Derakhshani Hamadani,[1] Fahmida Tofail,[1] Kathleen M. Rasmussen,[4] and Marie Vahter[3]

1. International Centre for Diarrhoeal Disease Research, Bangladesh, Dhaka, Bangladesh;
2. International Food Policy Research Institute, Washington, DC, USA;
3. Institute of Environmental Medicine, Karolinska Institutet, Stockholm, Sweden;
4. Division of Nutritional Sciences, Cornell University, Ithaca, NY, USA.

Corresponding author:
Professor
Marie Vahter, PhD
Institute of Environmental Medicine
Karolinska Institutet
Box 210, S-171 77 Stockholm, Sweden
E-mail: Marie.Vahter@ki.se
Phone: +46 8 52487540, Fax: +46 8 336981

Running title: Early Life Arsenic Exposure and Child Body Size

Table of Contents

Supplemental Table 1. Attained child *weight (kg)* at 3, 6, 9, 12, 18, and 24 months of age by quintiles of maternal urinary arsenic (U-As, µg/L) at 8 and 30 weeks of pregnancy. Data are presented for boys and girls.

Supplemental Table 2. Attained child *length (cm)* at 3, 6, 9, 12, 18, and 24 months of age by quintiles of maternal urinary arsenic (U-As, µg/L) at 8 and 30 weeks of pregnancy. Data are presented for boys and girls.

Supplemental Table 3. Multiple-adjusted linear regression analysis for evaluation of associations between quintiles of maternal urinary arsenic (U-As, µg/L) at 8 and 30 weeks of pregnancy and attained *weight (kg)* of children at 3, 6, 9, 12, 18, and 24 months of age. Data are presented for boys and girls.

Supplemental Table 4. Multiple-adjusted linear regression analysis for evaluation of associations between quintiles of maternal urinary arsenic (U-As, µg/L) at 8 and 30 weeks of pregnancy and attained *length (cm)* of children at 3, 6, 9, 12, 18, and 24 months of age. Data are presented for boys and girls.

Supplemental Table 5. Attained child *weight (kg)* at 18, 21, and 24 months of age by quintiles of child urinary arsenic (U-As, µg/L) at 18 months of age. Data are presented for all children, boys and girls.

Supplemental Table 6. Attained child *length (cm)* at 18, 21, and 24 months of age by quintiles of child urinary arsenic (U-As, µg/L) at 18 months of age. Data are presented for all children, boys and girls.

Supplemental Table 7. Odds ratio (OR) and 95% confidence interval (CI) for *underweight* in children at 18, 21, and 24 months of age, in relation to quintiles of child urinary arsenic (U-As, µg/L) at 18 months of age. Data are presented for all children, boys and girls.

Supplemental Table 8. Odds ratio (OR) and 95% confidence interval (CI) for *stunting* in children at 18, 21, and 24 months of age, in relation to quintiles of child urinary arsenic (U-As, µg/L) at 18 months of age. Data are presented for all children, boys and girls.

Supplemental Figure 1. Percent *underweight* (weight for age Z-score <-2) (A) and *stunting* (length for age Z-score <-2) (B) in boys and girls at 24 months of age by categorized urinary arsenic (median split, 34.2 µg/L) at 18 months.

Supplemental Table 1. Attained child *weight (kg)* at 3, 6, 9, 12, 18, and 24 months of age by quintiles of maternal urinary arsenic (U-As, µg/L) at 8 and 30 weeks of pregnancy. Data are presented for boys and girls.

Quintiles of maternal U-As; Median (range), µg/L	3 months Mean±SD	6 months Mean±SD	9 months Mean±SD	12 months Mean±SD	18 months Mean±SD	24 months Mean±SD
**At 8 weeks of pregnancy**						
Boys						
Overall mean weight	*5.51±0.72*	*7.01±0.88*	*7.78±0.98*	*8.30±1.04*	*9.19±1.14*	*10.0±1.22*
Q1: 23 (1.2-33)	5.49±0.76	7.05±0.87	7.84±1.01	8.42±1.16	9.35±1.32	10.2±1.41
Q2: 41 (33-57)	5.58±0.69	7.11±0.83	7.88±0.94	8.37±1.05	9.27±1.08	10.1±1.22
Q3: 80 (57-115)	5.39±0.75	6.90±0.93	7.66±0.97	8.15±0.97	9.01±1.02	9.88±1.15
Q4: 169 (116-245)	5.55±0.69	6.98±0.89	7.68±1.02	8.21±1.00	9.12±1.16	9.96±1.17
Q5: 378 (246-1611)	5.54±0.68	7.01±0.88	7.81±0.96	8.36±0.99	9.19±1.07	10.1±1.10
p-value for trend[a]	0.51	0.57	0.74	0.97	0.42	0.59
Girls						
Overall mean weight	*5.06±0.64*	*6.43±0.80*	*7.13±0.93*	*7.65±1.02*	*8.54±1.05*	*9.38±1.11*
Q1: 23 (1.2-33)	5.06±0.58	6.53±0.81	7.21±1.00	7.77±1.05	8.64±1.08	9.48±1.09
Q2: 41 (33-57)	5.15±0.68	6.51±0.84	7.18±0.95	7.69±1.14	8.54±1.19	9.38±1.21
Q3: 80 (57-115)	5.03±0.67	6.39±0.80	7.13±0.93	7.64±1.00	8.55±1.01	9.39±1.11
Q4: 169 (116-245)	5.09±0.61	6.36±0.79	7.11±0.88	7.59±0.96	8.48±0.96	9.34±1.03
Q5: 378 (246-1611)	4.99±0.63	6.37±0.76	7.02±0.89	7.58±0.93	8.47±0.98	9.32±1.09
p-value for trend[a]	0.11	0.066	0.045	0.10	0.18	0.26

[a] Linear trends across categories were tested using the median U-As concentrations within categories as continuous variable

Supplemental Table 1. *Cont.*

Quintiles of maternal U-As; Median (range), µg/L	3 months	6 months	9 months	12 months	18 months	24 months
	Mean±SD	Mean±SD	Mean±SD	Mean±SD	Mean±SD	Mean±SD
**At 30 weeks of pregnancy**						
Boys						
Overall mean weight	*5.52±0.70*	*7.03±0.88*	*7.79±0.98*	*8.31±1.04*	*9.21±1.14*	*10.0±1.23*
Q1: 25 (1.8-36)	5.58±0.73	7.05±0.90	7.86±1.01	8.37±1.15	9.32±1.32	10.2±1.46
Q2: 48 (36-63)	5.56±0.66	7.11±0.85	7.87±0.97	8.40±1.01	9.26±1.10	10.1±1.22
Q3: 84 (63-120)	5.50±0.71	7.03±0.88	7.77±0.96	8.26±1.10	9.18±1.12	9.98±1.19
Q4: 185 (121-272)	5.49±0.71	6.97±0.91	7.71±1.00	8.20±0.98	9.08±1.09	9.89±1.15
Q5: 414 (273-1632)	5.48±0.67	7.00±0.88	7.74±0.93	8.31±0.97	9.23±1.06	10.0±1.08
p-value for trend[a]	0.22	0.35	0.15	0.44	0.53	0.24
Girls						
Overall mean weight	*5.06±0.65*	*6.44±0.81*	*7.11±0.92*	*7.63±1.01*	*8.54±1.05*	*9.37±1.11*
Q1: 25 (1.8-36)	5.15±0.63	6.52±0.88	7.20±0.96	7.74±1.06	8.59±1.21	9.46±1.29
Q2: 48 (36-63)	5.09±0.66	6.44±0.80	7.12±1.02	7.64±1.01	8.47±1.03	9.35±1.05
Q3: 84 (63-120)	5.08±0.66	6.42±0.78	7.08±0.84	7.62±1.00	8.55±1.03	9.37±1.09
Q4: 185 (121-272)	5.02±0.57	6.47±0.79	7.16±0.90	7.66±1.06	8.61±1.01	9.41±1.08
Q5: 414 (273-1632)	4.96±0.70	6.34±0.78	6.97±0.85	7.51±0.89	8.47±0.96	9.28±1.04
p-value for trend[a]	0.011	0.086	0.038	0.048	0.53	0.19

[a] Linear trends across categories were tested using the median U-As concentrations within categories as continuous variable

Supplemental Table 2. Attained child *length (cm)* at 3, 6, 9, 12, 18, and 24 months of age by quintiles of maternal urinary arsenic (U-As, µg/L) at 8 and 30 weeks of pregnancy. Data are presented for boys and girls.

Quintiles of maternal U-As; Median (range), µg/L	3 months<br>Mean±SD	6 months<br>Mean±SD	9 months<br>Mean±SD	12 months<br>Mean±SD	18 months<br>Mean±SD	24 months<br>Mean±SD
**At 8 weeks of pregnancy**						
Boys						
Overall mean length	*58.5±2.3*	*64.5±2.4*	*68.5±2.5*	*71.6±2.6*	*76.8±3.0*	*81.3±3.4*
Q1: 23 (1.2-33)	58.5±2.5	64.7±2.4	68.8±2.4	71.9±2.7	77.1±3.1	81.5±3.4
Q2: 41 (33-57)	58.6±2.3	64.6±2.3	68.5±2.1	71.7±2.3	76.7±2.7	81.4±3.0
Q3: 80 (57-115)	58.2±2.2	64.2±2.5	68.2±2.6	71.3±2.7	76.3±3.1	80.9±3.5
Q4: 169 (116-245)	58.5±2.4	64.2±2.5	68.3±2.8	71.5±2.9	76.7±3.4	81.1±3.6
Q5: 378 (246-1611)	58.6±2.2	64.7±2.3	68.7±2.2	71.7±2.5	77.0±2.9	81.5±3.3
p-value for trend[a]	0.57	0.69	0.65	0.79	0.37	0.76
Girls						
Overall mean length	*57.2±2.0*	*62.7±2.2*	*66.8±2.3*	*69.9±2.5*	*75.0±2.9*	*79.7±3.1*
Q1: 23 (1.2-33)	57.1±2.0	62.9±2.3	66.9±2.6	70.2±2.7	75.3±3.2	80.0±3.3
Q2: 41 (33-57)	57.4±2.1	62.7±2.1	66.7±2.2	69.8±2.4	74.9±2.9	79.6±3.0
Q3: 80 (57-115)	57.0±2.0	62.7±2.2	66.8±2.3	70.1±2.5	75.1±2.8	79.7±3.2
Q4: 169 (116-245)	57.2±2.1	62.5±2.3	66.8±2.4	69.7±2.4	74.9±2.9	79.4±3.2
Q5: 378 (246-1611)	57.1±2.0	62.7±1.9	66.7±2.1	69.6±2.3	74.9±2.6	79.7±2.8
p-value for trend[a]	0.65	0.40	0.53	0.037	0.33	0.59

[a] Linear trends across categories were tested using the median U-As concentrations within categories as continuous variable

Supplemental Table 2. *Cont.*

Quintiles of maternal U-As; Median (range), µg/L	3 months	6 months	9 months	12 months	18 months	24 months
	Mean±SD	Mean±SD	Mean±SD	Mean±SD	Mean±SD	Mean±SD
**At 30 weeks of pregnancy**						
Boys						
Overall mean length	*58.5±2.3*	*64.5±2.4*	*68.9±2.43*	*71.7±2.6*	*76.8±3.0*	*81.3±3.3*
Q1: 25 (1.8-36)	58.9±2.3	64.7±2.4	68.8±2.4	72.0±2.6	77.1±3.0	81.6±3.4
Q2: 48 (36-63)	58.7±2.3	64.6±2.3	68.7±2.5	71.8±2.6	76.8±3.0	81.4±3.3
Q3: 84 (63-120)	58.4±2.5	64.5±2.4	68.6±2.4	71.5±2.6	76.6±2.9	81.2±3.4
Q4: 185 (121-272)	58.2±2.3	64.3±2.4	68.3±2.6	71.5±2.6	76.5±3.1	81.1±3.3
Q5: 414 (273-1632)	58.4±2.1	64.5±2.4	68.5±2.3	71.6±2.5	76.9±3.0	81.4±3.2
p-value for trend[a]	0.13	0.37	0.28	0.24	0.76	0.72
Girls						
Overall mean length	*57.2±2.1*	*62.7±2.2*	*66.8±2.3*	*69.9±2.5*	*75.0±2.9*	*79.7±3.1*
Q1: 25 (1.8-36)	57.4±2.1	63.1±2.3	66.9±2.3	70.2±2.6	75.3±2.0	80.1±3.5
Q2: 48 (36-63)	57.1±2.1	62.7±2.2	66.7±2.5	69.7±2.5	74.9±3.0	79.5±3.1
Q3: 84 (63-120)	57.1±2.0	62.8±2.0	66.8±2.2	69.9±2.5	75.2±3.0	79.7±3.2
Q4: 185 (121-272)	57.3±1.9	62.9±2.0	67.2±2.1	70.1±2.4	75.3±2.7	79.7±3.0
Q5: 414 (273-1632)	56.9±2.2	62.3±2.3	66.5±2.3	69.5±2.4	74.6±2.7	79.4±2.8
p-value for trend[a]	0.071	0.004	0.19	0.044	0.17	0.12

[a] Linear trends across categories were tested using the median U-As concentrations within categories as continuous variable

Supplemental Table 3. Multiple-adjusted linear regression analysis for evaluation of associations between quintiles of maternal urinary arsenic (U-As, µg/L) at 8 and 30 weeks of pregnancy and attained *weight (kg)* of children at 3, 6, 9, 12, 18, and 24 months of age. Data are presented for boys and girls.

Quintiles of maternal U-As; Median (range), µg/L	3 months β (95% CI)	6 months β (95% CI)	9 months β (95% CI)	12 months β (95% CI)	18 months β (95% CI)	24 months β (95% CI)
**At 8 weeks of pregnancy**						
Boys						
Unadjusted[a]						
Q1: 23 (1.2-33)	Reference	Reference	Reference	Reference	Reference	Reference
Q2: 41 (33-57)	0.096 (-0.061; 0.25)	0.058 (-0.12; 0.24)	0.049 (-0.15; 0.25)	-0.051 (-0.25; 0.15)	-0.084 (-0.31; 0.14)	-0.036 (-0.28; 0.20)
Q3: 80 (57-115)	-0.083 (-0.24; 0.075)	-0.14 (-0.32; 0.04)	-0.18 (-0.38; 0.023)	-0.27 (-0.47; -0.072)	-0.34 (-0.56; -0.12)	-0.28 (-0.52; -0.041)
Q4: 169 (116-245)	0.057 (-0.11; 0.22)	-0.060 (-0.24; 0.12)	-0.16 (-0.36; 0.041)	-0.21 (-0.41; -0.002)	-0.23 (-0.46; -0.009)	-0.21 (-0.45; 0.032)
Q5: 378 (246-1611)	0.070 (-0.092; 0.23)	-0.043 (-0.23; 0.14)	-0.030 (-0.23; 0.17)	-0.062 (-0.26; 0.14)	-0.17 (-0.39; 0.054)	-0.11 (-0.35; 0.13)
Adjusted[b]						
Q1: 23 (1.2-33)	Reference	Reference	Reference	Reference	Reference	Reference
Q2: 41 (33-57)	0.11 (-0.04; 0.26)	0.10 (-0.071; 0.27)	0.077 (-0.11; 0.26)	-0.018 (-0.20; 0.17)	-0.030 (-0.24; 0.18)	0.001 (-0.22; 0.22)
Q3: 80 (57-115)	-0.034 (-0.18; 0.12)	-0.079 (-0.25; 0.094)	-0.11 (-0.30; 0.073)	-0.20 (-0.38; -0.011)	-0.23 (-0.43; -0.024)	-0.18 (-0.41; 0.039)
Q4: 169 (116-245)	0.13 (-0.028; 0.28)	0.043 (-0.13; 0.22)	-0.048 (-0.24; 0.14)	-0.088 (-0.28; 0.10)	-0.086 (-0.29; 0.12)	-0.082 (-0.31; 0.14)
Q5: 378 (246-1611)	0.17 (0.017; 0.33)	0.084 (-0.092; 0.26)	0.086 (-0.10; 0.27)	0.080 (-0.11; 0.27)	0.015 (-0.19; 0.22)	0.066 (-0.16; 0.29)

[a] Urinary arsenic (quintiles of U-As at 8 and 30 weeks of gestation) and actual age in respective age group were entered

[b] Urinary arsenic, age, maternal BMI (continuous variable) and SES (quintiles as continuous variable) were entered

Supplemental Table 3. *Cont.*

Quintiles of maternal U-As; Median (range), µg/L	3 months β (95% CI)	6 months β (95% CI)	9 months β (95% CI)	12 months β (95% CI)	18 months β (95% CI)	24 months β (95% CI)
**At 8 weeks of pregnancy**						
Girls						
Unadjusted[a]						
Q1: 23 (1.2-33)	Reference	Reference	Reference	Reference	Reference	Reference
Q2: 41 (33-57)	0.080 (-0.068; 0.23)	-0.010 (-0.19; 0.17)	-0.037 (-0.23; 0.16)	-0.077 (-0.28; 0.13)	-0.10 (-0.32; 0.11)	-0.096 (-0.33; 0.14)
Q3: 80 (57-115)	-0.043 (-0.19; 0.10)	-0.14 (-0.31; 0.028)	-0.088 (-0.28; 0.11)	-0.13 (-0.34; 0.074)	-0.095 (-0.31; 0.12)	-0.084 (-0.31; 0.14)
Q4: 169 (116-245)	0.023 (-0.13; 0.18)	-0.16 (-0.34; 0.017)	-0.11 (-0.30; 0.085)	-0.18 (-0.39; 0.026)	-0.16 (-0.37; 0.052)	-0.14 (-0.37; 0.088)
Q5: 378 (246-1611)	-0.079 (-0.23; 0.071)	-0.16 (-0.33; 0.013)	-0.19 (-0.39; 0.002)	-0.19 (-0.40; 0.017)	-0.16 (-0.37; 0.058)	-0.15 (-0.38; 0.077)
Adjusted[b]						
Q1: 23 (1.2-33)	Reference	Reference	Reference	Reference	Reference	Reference
Q2: 41 (33-57)	0.10 (-0.042; 0.24)	0.032 (-0.14; 0.20)	0.009 (-0.18; 0.19)	-0.030 (-0.22; 0.16)	-0.035 (-0.23; 0.16)	-0.033 (-0.25; 0.18)
Q3: 80 (57-115)	-0.007 (-0.15; 0.13)	-0.085 (-0.25; 0.078)	-0.008 (-0.19; 0.18)	-0.056 (-0.25; 0.14)	0.003 (-0.20; 0.20)	0.007 (-0.21; 0.22)
Q4: 169 (116-245)	0.048 (-0.10; 0.20)	-0.10 (-0.27; 0.066)	-0.038 (-0.22; 0.15)	-0.094 (-0.29; 0.098)	-0.038 (-0.24; 0.16)	-0.031 (-0.25; 0.18)
Q5: 378 (246-1611)	-0.021 (-0.17; 0.12)	-0.060 (-0.23; 0.11)	-0.054 (-0.24; 0.13)	-0.035 (-0.23; 0.16)	0.034 (-0.17; 0.24)	0.024 (-0.19; 0.24)

[a] Urinary arsenic (quintiles of U-As at 8 and 30 weeks of gestation) and actual age in respective age group were entered
[b] Urinary arsenic, age, maternal BMI (continuous variable) and SES (quintiles as continuous variable) were entered

Supplemental Table 3. *Cont.*

Quintiles of maternal U-As; Median (range), µg/L	3 months β (95% CI)	6 months β (95% CI)	9 months β (95% CI)	12 months β (95% CI)	18 months β (95% CI)	24 months β (95% CI)
**At 30 weeks of pregnancy**						
Boys						
Unadjusted[a]						
Q1: 25 (1.8-36)	Reference	Reference	Reference	Reference	Reference	Reference
Q2: 48 (36-63)	-0.016 (-0.17; 0.14)	0.068 (-0.12; 0.25)	0.015 (-0.19; 0.22)	0.034 (-0.17; 0.23)	-0.062 (-0.28; 0.16)	-0.018 (-0.26; 0.22)
Q3: 84 (63-120)	-0.081 (-0.24; 0.075)	-0.006 (-0.19; 0.18)	-0.079 (-0.28; 0.12)	-0.10 (-0.30; 0.096)	-0.13 (-0.35; 0.087)	-0.17 (-0.41; 0.062)
Q 4:185 (121-272)	-0.088 (-0.25; 0.071)	-0.074 (-0.26; 0.11)	-0.15 (-0.35; 0.045)	-0.17 (-0.37; 0.030)	-0.23 (-0.45; -0.009)	-0.26 (-0.50; -0.026)
Q5: 414 (273-1632)	-0.092 (-0.26; 0.071)	-0.042 (-0.23; 0.15)	-0.12 (-0.32; 0.086)	-0.057 (-0.26; 0.15)	-0.081 (-0.31; 0.14)	-0.14 (-0.38; 0.10)
Adjusted[b]						
Q1: 25 (1.8-36)	Reference	Reference	Reference	Reference	Reference	Reference
Q2: 48 (36-63)	0.050 (-0.10; 0.20)	0.13 (-0.044; 0.31)	0.086 (-0.10; 0.28)	0.090 (-0.096; 0.28)	0.014 (-0.19; 0.22)	0.052 (-0.17; 0.28)
Q3: 84 (63-120)	-0.032 (-0.18; 0.12)	0.040 (-0.14; 0.22)	-0.040 (-0.23; 0.15)	-0.074 (-0.26; 0.11)	-0.086 (-0.29; 0.12)	-0.13 (-0.35; 0.095)
Q 4:185 (121-272)	-0.011 (-0.16; 0.14)	-0.003 (-0.18; 0.17)	-0.058 (-0.24; 0.13)	-0.11 (-0.29; 0.080)	-0.14 (-0.34; 0.068)	-0.18 (-0.40; 0.045)
Q5: 414 (273-1632)	0.013 (-0.15; 0.17)	0.075 (-0.11; 0.26)	-0.002 (-0.19; 0.19)	0.052 (-0.14; 0.24)	0.061 (-0.15; 0.27)	0.012 (-0.22; 0.24)

[a] Urinary arsenic (quintiles of U-As at 8 and 30 weeks of gestation) and actual age in respective age group were entered
[b] Urinary arsenic, age, maternal BMI (continuous variable) and SES (quintiles as continuous variable) were entered

Supplemental Table 3. *Cont.*

Quintiles of maternal U-As; Median (range), µg/L	3 months β (95% CI)	6 months β (95% CI)	9 months β (95% CI)	12 months β (95% CI)	18 months β (95% CI)	24 months β (95% CI)
**At 30 weeks of pregnancy**						
Girls						
Unadjusted[a]						
Q1: 25 (1.8-36)	Reference	Reference	Reference	Reference	Reference	Reference
Q2: 48 (36-63)	-0.064 (-0.22; 0.088)	-0.075 (-0.25; 0.099)	-0.079 (-0.27; 0.11)	-0.095 (-0.29; 0.10)	-0.12 (-0.33; 0.093)	-0.11 (-0.33; 0.12)
Q3: 84 (63-120)	-0.098 (-0.25; 0.057)	-0.082 (-0.26; 0.095)	-0.12 (-0.31; 0.074)	-0.12 (-0.32; 0.081)	-0.048 (-0.26; 0.16)	-0.089 (-0.32; 0.14)
Q 4:185 (121-272)	-0.15 (-0.31; 0.005)	-0.040 (-0.22; 0.14)	-0.039 (-0.23; 0.15)	-0.078 (-0.28; 0.12)	0.026 (-0.19; 0.24)	-0.053 (-0.28; 0.17)
Q5: 414 (273-1632)	-0.21 (-0.37; -0.061)	-0.17 (-0.34; 0.005)	-0.23 (-0.42; -0.038)	-0.23 (-0.42; -0.031)	-0.12 (-0.33; 0.089)	-0.18 (-0.40; 0.040)
Adjusted[b]						
Q1: 25 (1.8-36)	Reference	Reference	Reference	Reference	Reference	Reference
Q2: 48 (36-63)	-0.054 (-0.20; 0.093)	-0.020 (-0.19; 0.15)	-0.045 (-0.22; 0.13)	-0.054 (-0.24; 0.13)	-0.044 (-0.24; 0.15)	-0.052 (-0.26; 0.16)
Q3: 84 (63-120)	-0.097 (-0.25; 0.052)	-0.068 (-0.24; 0.10)	-0.15 (-0.33; 0.031)	-0.14 (-0.33; 0.045)	-0.043 (-0.24; 0.15)	-0.087 (-0.30; 0.13)
Q 4:185 (121-272)	-0.13 (-0.28; 0.026)	0.005 (-0.17; 0.18)	0.012 (-0.17; 0.19)	-0.018 (-0.20; 0.17)	0.12 (-0.077; 0.31)	0.023 (-0.19; 0.23)
Q5: 414 (273-1632)	-0.16 (-0.31; -0.015)	-0.066 (-0.23; 0.10)	-0.12 (-0.30; 0.056)	-0.10 (-0.29; 0.079)	0.033 (-0.16; 0.23)	-0.043 (-0.25; 0.17)

[a] Urinary arsenic (quintiles of U-As at 8 and 30 weeks of gestation) and actual age in respective age group were entered
[b] Urinary arsenic, age, maternal BMI (continuous variable) and SES (quintiles as continuous variable) were entered

Supplemental Table 4. Multiple-adjusted linear regression analysis for evaluation of associations between quintiles of maternal urinary arsenic (U-As, µg/L) at 8 and 30 weeks of pregnancy and attained *length (cm)* of children at 3, 6, 9, 12, 18, and 24 months of age. Data are presented for boys and girls.

Quintiles of maternal U-As; Median (range), µg/L	3 months	6 months	9 months	12 months	18 months	24 months
	β (95% CI)	β (95% CI)	β (95% CI)	β (95% CI)	β (95% CI)	β (95% CI)
**At 8 weeks of pregnancy**						
Boys						
Unadjusted[a]						
Q1: 23 (1.2-33)	Reference	Reference	Reference	Reference	Reference	Reference
Q2: 41 (33-57)	0.18 (-0.34; 0.69)	-0.11 (-0.60; 0.38)	-0.21 (-0.71; 0.29)	-0.25 (-0.76; 0.25)	-0.44 (-1.03; 0.15)	-0.063 (-0.72; 0.60)
Q3: 80 (57-115)	-0.19 (-0.71; 0.33)	-0.42 (-0.91; 0.078)	-0.57 (-1.07;-0.072)	-0.64 (-1.14;-0.14)	-0.78 (-1.36; -0.19)	-0.62 (-1.28; 0.044)
Q4: 169 (116-245)	-0.037 (-0.57; 0.50)	-0.41 (-0.91; 0.094)	-0.43 (-0.93; 0.068)	-0.46 (-0.97; 0.053)	-0.40 (-1.00; 0.19)	-0.42 (-1.08; 0.25)
Q5: 378 (246-1611)	0.20 (-0.34; 0.73)	0.019 (-0.48; 0.52)	-0.049 (-0.54; 0.45)	-0.26 (-0.76; 0.24)	-0.086 (-0.67; 0.51)	-0.030 (-0.70; 0.64)
Adjusted[b]						
Q1: 23 (1.2-33)	Reference	Reference	Reference	Reference	Reference	Reference
Q2: 41 (33-57)	0.18 (-0.32; 0.68)	-0.049 (-0.53; 0.43)	-0.19 (-0.67; 0.29)	-0.23 (-0.70; 0.25)	-0.36 (-0.91; 0.19)	-0.012 (-0.63; 0.61)
Q3: 80 (57-115)	-0.088 (-0.60; 0.42)	-0.29 (-0.78; 0.19)	-0.49 (-0.97;-0.013)	-0.50 (-0.98; -0.030)	-0.52 (-1.07; 0.025)	-0.38 (-1.01; 0.24)
Q4: 169 (116-245)	0.12 (-0.41; 0.64)	-0.18 (-0.67; 0.31)	-0.23 (-0.71; 0.25)	-0.20 (-0.68; 0.29)	-0.035 (-0.59; 0.52)	-0.061 (-0.68; 0.56)
Q5: 378 (246-1611)	0.41 (-0.12; 0.93)	0.28 (-0.21; 0.77)	0.15 (-0.33; 0.63)	-0.006 (-0.48; 0.47)	0.32 (-0.23; 0.88)	0.40 (-0.22; 1.03)

[a] Urinary arsenic (quintiles of U-As at 8 and 30 weeks of gestation) and actual age in respective age group were entered

[b] Urinary arsenic, age, maternal BMI (continuous variable) and SES (quintiles as continuous variable) were entered

Supplemental Table 4. *Cont.*

Quintiles of maternal U-As; Median (range), µg/L	3 months β (95% CI)	6 months β (95% CI)	9 months β (95% CI)	12 months β (95% CI)	18 months β (95% CI)	24 months β (95% CI)
**At 8 weeks of pregnancy**						
Girls						
Unadjusted[a]						
Q1: 23 (1.2-33)	Reference	Reference	Reference	Reference	Reference	Reference
Q2: 41 (33-57)	0.17 (-0.030; 0.65)	-0.15 (-0.63; 0.32)	-0.14 (-0.63; 0.36)	-0.31 (-0.82; 0.19)	-0.34 (-0.92; 0.25)	-0.38 (-1.03; 0.27)
Q3: 80 (57-115)	-0.13 (-0.60; 0.35)	-0.20 (-0.66; 0.26)	-0.079 (-0.57; 0.41)	-0.071 (-0.58; 0.44)	-0.20 (-0.78; 0.39)	-0.31 (-0.96; 0.33)
Q4: 169 (116-245)	-0.006 (-0.50; 0.15)	-0.38 (-0.86; 0.020)	-0.069 (-0.56; 0.42)	-0.45 (-0.96; 0.055)	-0.41 (-0.99; 0.18)	-0.57 (-1.21; 0.064)
Q5: 378 (246-1611)	-0.042 (-0.52; 0.44)	-0.23 (-0.69; 0.24)	-0.21 (-0.70; 0.29)	-0.54 (-1.04; -0.030)	-0.33 (-0.92; 0.27)	-0.31 (-0.95; 0.34)
Adjusted[b]						
Q1: 23 (1.2-33)	Reference	Reference	Reference	Reference	Reference	Reference
Q2: 41 (33-57)	0.24 (-0.23; 0.71)	-0.045 (-0.50; 0.42)	0.002 (-0.47; 0.47)	-0.20 (-0.68; 0.28)	-0.15 (-0.71; 0.40)	-0.19 (-0.80; 0.43)
Q3: 80 (57-115)	-0.044 (-0.51; 0.42)	-0.080 (-0.53; 0.37)	0.12 (-0.35; 0.59)	0.11 (-0.37; 0.59)	0.053 (-0.50; 0.61)	-0.035 (-0.64; 0.57)
Q4: 169 (116-245)	0.076 (-0.42; 0.57)	-0.23 (-0.69; 0.24)	0.12 (-0.35; 0.59)	-0.23 (-0.71; 0.25)	-0.072 (-0.63; 0.48)	-0.23 (-0.83; 0.38)
Q5: 378 (246-1611)	0.11 (-0.36; 0.59)	0.014 (-0.44; 0.47)	0.13 (-0.34; 0.61)	-0.17 (-0.65; 0.31)	0.19 (-0.38; 0.75)	0.17 (-0.44; 0.79)

[a] Urinary arsenic (quintiles of U-As at 8 and 30 weeks of gestation) and actual age in respective age group were entered

[b] Urinary arsenic, age, maternal BMI (continuous variable) and SES (quintiles as continuous variable) were entered

Supplemental Table 4. *Cont.*

Quintiles of maternal U-As; Median (range), µg/L	3 months β (95% CI)	6 months β (95% CI)	9 months β (95% CI)	12 months β (95% CI)	18 months β (95% CI)	24 months β (95% CI)
**At 30 weeks of pregnancy**						
Boys						
Unadjusted[a]						
Q1: 25 (1.8-36)	Reference	Reference	Reference	Reference	Reference	Reference
Q2: 48 (36-63)	-0.12 (-0.63; 0.39)	-0.12 (-0.62; 0.37)	-0.016 (-0.51; 0.48)	-0.16 (-0.65; 0.34)	-0.32 (-0.90; 0.27)	-0.18 (-0.83; 0.47)
Q3: 84 (63-120)	-0.52 (-1.04; -0.010)	-0.028 (-0.64; 0.35)	-0.15 (-0.65; 0.34)	-0.45 (-0.95; 0.039)	-0.49 (-1.07; 0.089)	-0.43 (-1.07; 0.21)
Q4: 185 (121-272)	-0.74 (-1.26; -0.22)	-0.046 (-0.92; 0.069)	-0.43 (-0.92; 0.052)	-0.52 (-1.01; -0.025)	-0.60 (-1.18; -0.014)	-0.49 (-1.13; 0.15)
Q5: 414 (273-1632)	-0.39 (-0.92; 0.15)	-0.13 (-0.75; 0.28)	-0.20 (-0.70; 0.31)	-0.37 (-0.87; 0.13)	-0.22 (-0.82; 0.37)	-0.21 (-0.88; 0.45)
Adjusted[b]						
Q1: 25 (1.8-36)	Reference	Reference	Reference	Reference	Reference	Reference
Q2: 48 (36-63)	-0.058 (-0.44; 0.56)	0.082 (-0.40; 0.57)	0.13 (-0.35; 0.61)	-0.014 (-0.48; 0.46)	-0.095 (-0.65; 0.46)	0.040 (-0.57; 0.65)
Q3: 84 (63-120)	-0.40 (-0.90; 0.11)	-0.023 (-0.51; 0.46)	-0.092 (-0.57; 0.38)	-0.41 (-0.87; 0.061)	-0.37 (-0.91; 0.17)	-0.29 (-0.89; 0.32)
Q4: 185 (121-272)	-0.55 (-1.06; -0.031)	-0.23 (-0.71; 0.26)	-0.27 (-0.74; 0.20)	-0.37 (-0.84; 0.096)	-0.37 (-0.92; 0.17)	-0.25 (-0.85; 0.36)
Q5: 414 (273-1632)	-0.16 (-0.69; 0.37)	0.039 (-0.46; 0.54)	0.004 (-0.48; 0.49)	-0.17 (-0.65; 0.31)	0.11 (-0.45; 0.67)	0.16 (-0.47; 0.78)

[a] Urinary arsenic (quintiles of U-As at 8 and 30 weeks of gestation) and actual age in respective age group were entered
[b] Urinary arsenic, age, maternal BMI (continuous variable) and SES (quintiles as continuous variable) were entered

Supplemental Table 4. *Cont.*

Quintiles of maternal U-As; Median (range), µg/L	3 months β (95% CI)	6 months β (95% CI)	9 months β (95% CI)	12 months β (95% CI)	18 months β (95% CI)	24 months β (95% CI)
**At 30 weeks of pregnancy**						
Girls						
Unadjusted[a]						
Q1: 25 (1.8-36)	Reference	Reference	Reference	Reference	Reference	Reference
Q2: 48 (36-63)	-0.29 (-0.77; 0.19)	-0.30 (-0.77; 0.17)	-0.19 (-0.67; 0.29)	-0.45 (-0.95; 0.039)	-0.15 (-0.73; 0.43)	-0.58 (-1.22; 0.049)
Q3: 84 (63-120)	-0.39 (-0.88; 0.094)	-0.23 (-0.71; 0.24)	-0.088 (-0.58; 0.40)	-0.26 (-0.76; 0.24)	0.13 (-0.46; 0.71)	-0.41 (-1.05; 0.23)
Q4: 185 (121-272)	-0.22 (-0.71; 0.28)	-0.12 (-0.60; 0.36)	0.29 (-0.19; 0.77)	-0.098 (-0.60; 0.40)	0.26 (-0.32; 0.85)	-0.40 (-1.03; 0.24)
Q5: 414 (273-1632)	-0.61 (-1.10; -0.13)	-0.71 (-1.17; -0.24)	-0.40 (-0.88; 0.082)	-0.64 (-1.13; -0.15)	-0.40 (-0.98; 0.17)	-0.71 (-1.33; -0.084)
Adjusted[b]						
Q1: 25 (1.8-36)	Reference	Reference	Reference	Reference	Reference	Reference
Q2: 48 (36-63)	-0.27 (-0.74; 0.21)	-0.16 (-0.62; 0.30)	-0.078 (-0.54; 0.38)	-0.33 (-0.80; 0.14)	0.045 (-0.50; 0.59)	-0.39 (-0.99; 0.21)
Q3: 84 (63-120)	-0.40 (-0.88; 0.081)	-0.17 (-0.64; 0.29)	-0.12 (-0.59; 0.35)	-0.27 (-0.75; 0.20)	0.19 (-0.37; 0.74)	-0.33 (-0.94; 0.28)
Q4: 185 (121-272)	-0.16 (-0.65; 0.33)	-0.007 (-0.47; 0.46)	0.42 (-0.039; 0.88)	0.066 (-0.41; 0.54)	0.50 (-0.052; 1.05)	-0.15 (-0.75; 0.46)
Q5: 414 (273-1632)	-0.50 (-0.98; -0.025)	-0.49 (-0.94; -0.030)	-0.16 (-0.62; 0.30)	-0.34 (-0.81; 0.13)	-0.007 (-0.56; 0.54)	-0.32 (-0.91; 0.28)

[a] Urinary arsenic (quintiles of U-As at 8 and 30 weeks of gestation) and actual age in respective age group were entered

[b] Urinary arsenic, age, maternal BMI (continuous variable) and SES (quintiles as continuous variable) were entered

Supplemental Table 5. Attained child *weight (kg)* at 18, 21, and 24 months of age by quintiles of child urinary arsenic (U-As, µg/L) at 18 months of age. Data are presented for all children, boys and girls.

Quintiles of U-As at 18 months; Median (range), µg/L	18 months Mean±SD	21 months Mean±SD	24 months Mean±SD
All children			
Overall mean weight	*8.88±1.15*	*9.28±1.18*	*9.72±1.23*
Q1: 12 (2.4-16)	9.10±1.26	9.46±1.30	9.90±1.39
Q2: 20 (16-26)	8.86±1.06	9.31±1.20	9.71±1.20
Q3: 34 (26-46)	8.81±1.13	9.22±1.13	9.64±1.18
Q4: 64 (46-96)	8.75±1.11	9.17±1.10	9.62±1.16
Q5: 159 (96-937)	8.88±1.13	9.24±1.14	9.73±1.22
p-value for trend[a]	0.21	0.056	0.42
Boys			
Overall mean weight	*9.20±1.14*	*9.59±1.18*	*10.0±1.25*
Q1: 12 (2.4-16)	9.32±1.20	9.66±1.33	10.1±1.43
Q2: 20 (16-26)	9.14±1.04	9.61±1.15	10.0±1.17
Q3: 34 (26-46)	9.18±1.16	9.54±1.16	10.0±1.22
Q4: 64 (46-96)	9.11±1.07	9.51±1.08	9.97±1.15
Q5: 159 (96-937)	9.23±1.18	9.62±1.15	10.1±1.25
p-value for trend[a]	0.98	0.99	0.67
Girls			
Overall mean weight	*8.54±1.06*	*8.94±1.08*	*9.38±1.12*
Q1: 12 (2.4-16)	8.86±1.26	9.22±1.22	9.65±1.30
Q2: 20 (16-26)	8.57±1.00	9.01±1.16	9.42±1.15
Q3: 34 (26-46)	8.26±0.92	8.83±0.96	9.19±0.95
Q4: 64 (46-96)	8.38±1.02	8.81±1.01	9.28±1.05
Q5: 159 (96-937)	8.54±0.97	8.86±1.00	9.37±1.08
p-value for trend[a]	0.15	0.018	0.28

[a] Linear trends across categories were tested using the median U-As concentrations within categories as continuous variable

Supplemental Table 6. Attained child *length (cm)* at 18, 21, and 24 months of age by quintiles of child urinary arsenic (U-As, µg/L) at 18 months of age. Data are presented for all children, boys and girls.

Quintiles of U-As at 18 months; Median (range), µg/L	18 months Mean±SD	21 months Mean±SD	24 months Mean±SD
All children			
Overall mean length	*75.9±3.1*	*78.4±3.2*	*80.5±3.3*
Q1: 12 (2.4-16)	76.2±3.0	78.8±3.2	80.9±3.4
Q2: 20 (16-26)	76.0±3.1	78.4±3.3	80.7±3.4
Q3: 34 (26-46)	75.7±3.1	78.3±3.2	80.4±3.3
Q4: 64 (46-96)	75.8±3.1	78.1±3.2	80.1±3.2
Q5: 159 (96-937)	76.0±3.1	78.4±3.2	80.5±3.3
p-value for trend[a]	0.78	0.24	0.13
Boys			
Overall mean length	*76.8±3.0*	*79.2±3.2*	*81.3±3.4*
Q1: 12 (2.4-16)	76.8±2.9	79.3±3.2	81.4±3.4
Q2: 20 (16-26)	76.9±2.9	79.2±3.3	81.3±3.4
Q3: 34 (26-46)	76.5±3.1	79.0±3.2	81.2±3.3
Q4: 64 (46-96)	76.7±3.0	78.9±3.1	81.0±3.2
Q5: 159 (96-937)	77.0±3.2	79.3±3.3	81.4±3.4
p-value for trend[a]	0.38	0.70	0.91
Girls			
Overall mean length	*75.0±2.9*	*77.5±3.0*	*79.7±3.1*
Q1: 12 (2.4-16)	75.6±3.1	78.2±3.2	80.4±3.4
Q2: 20 (16-26)	75.0±3.0	77.6±3.1	80.0±3.2
Q3: 34 (26-46)	74.6±2.8	77.4±3.0	79.4±3.1
Q4: 64 (46-96)	74.8±2.9	77.2±2.9	79.3±2.9
Q5: 159 (96-937)	75.0±2.6	77.4±2.9	79.6±3.0
p-value for trend[a]	0.37	0.094	0.063

[a] Linear trends across categories were tested using the median U-As concentrations within categories as continuous variable

Supplemental Table 7. Odds ratio (OR) and 95% confidence interval (CI) for *underweight* in children at 18, 21, and 24 months of age, in relation to quintiles of child urinary arsenic (U-As, µg/L) at 18 months of age. Data are presented for all children, boys and girls.

Quintiles of U-As at 18 months; Median (range), µg/L	18 months OR (95% CI)	21 months OR (95% CI)	24 months OR (95% CI)
All children	*n* = 755 cases	*n* = 808 cases	*n* = 837 cases
Unadjusted[a]			
Q1: 12 (2.4-16)	1.00 (Reference)	1.00 (Reference)	1.00 (Reference)
Q2: 20 (16-26)	1.20 (0.90-1.59)	1.07 (0.81-1.41)	1.05 (0.80-1.39)
Q3: 34 (26-46)	1.26 (0.95-1.67)	1.14 (0.86-1.50)	1.28 (0.97-1.68)
Q4: 64 (46-96)	1.52 (1.15-2.00)	1.41 (1.07-1.85)	1.27 (0.96-1.67)
Q5: 159 (96-937)	1.18 (0.89-1.57)	1.07 (0.81-1.42)	1.12 (0.85-1.48)
Adjusted[b]			
Q1: 12 (2.4-16)	1.00 (Reference)	1.00 (Reference)	1.00 (Reference)
Q2: 20 (16-26)	1.03 (0.76-1.38)	0.92 (0.69-1.24)	0.91 (0.68-1.22)
Q3: 34 (26-46)	1.08 (0.81-1.45)	1.00 (0.75-1.34)	1.08 (0.81-1.45)
Q4: 64 (46-96)	1.28 (0.96-1.71)	1.18 (0.89-1.58)	1.06 (0.79-1.42)
Q5: 159 (96-937)	1.00 (0.74-1.34)	0.91 (0.67-1.22)	0.94 (0.70-1.26)
Boys	*n* = 401 cases	*n* = 427 cases	*n* = 452 cases
Unadjusted[a]			
Q1: 12 (2.4-16)	1.00 (Reference)	1.00 (Reference)	1.00 (Reference)
Q2: 20 (16-26)	1.15 (0.78-1.69)	0.86 (0.59-1.26)	0.98 (0.67-1.43)
Q3: 34 (26-46)	1.06 (0.73-1.55)	0.99 (0.69-1.43)	1.08 (0.75-1.56)
Q4: 64 (46-96)	1.35 (0.92-1.97)	1.15 (0.79-1.67)	1.19 (0.82-1.73)
Q5: 159 (96-937)	1.06 (0.72-1.57)	0.84 (0.57-1.24)	0.93 (0.64-1.37)
Adjusted[c]			
Q1: 12 (2.4-16)	1.00 (Reference)	1.00 (Reference)	1.00 (Reference)
Q2: 20 (16-26)	0.98 (0.65-1.46)	0.73 (0.49-1.08)	0.83 (0.56-1.23)
Q3: 34 (26-46)	0.87 (0.58-1.29)	0.81 (0.55-1.20)	0.90 (0.61-1.31)
Q4: 64 (46-96)	1.12 (0.75-1.67)	0.94 (0.64-1.39)	1.00 (0.68-1.49)
Q5: 159 (96-937)	0.92 (0.61-1.38)	0.73 (0.49-1.10)	0.79 (0.53-1.18)
Girls	*n* = 354 cases	*n* = 381 cases	*n* = 385 cases
Unadjusted[a]			
Q1: 12 (2.4-16)	1.00 (Reference)	1.00 (Reference)	1.00 (Reference)
Q2: 20 (16-26)	1.28 (0.84-1.94)	1.38 (0.92-2.09)	1.19 (0.79-1.80)
Q3: 34 (26-46)	1.56 (1.02-2.38)	1.36 (0.89-2.08)	1.60 (1.06-2.43)
Q4: 64 (46-96)	1.75 (1.16-2.64)	1.82 (1.21-2.73)	1.41 (0.93-2.11)
Q5: 159 (96-937)	1.36 (0.90-2.07)	1.42 (0.94-2.15)	1.41 (0.94-2.12)
Adjusted[c]			
Q1: 12 (2.4-16)	1.00 (Reference)	1.00 (Reference)	1.00 (Reference)
Q2: 20 (16-26)	1.10 (0.71-1.71)	1.24 (0.80-1.90)	1.04 (0.67-1.60)
Q3: 34 (26-46)	1.44 (0.93-2.24)	1.31 (0.84-2.04)	1.41 (0.91-2.19)
Q4: 64 (46-96)	1.50 (0.98-2.30)	1.57 (1.02-2.40)	1.15 (0.75-1.76)
Q5: 159 (96-937)	1.11 (0.72-1.73)	1.18 (0.76-1.82)	1.15 (0.75-1.78)

[a] Urinary arsenic (quintiles of U-As exposure at 18 months) and actual age in respective age group were entered

[b] Urinary arsenic, age, sex, maternal BMI (continuous variable) and SES (using quintiles as continuous variable) were entered

[c] Urinary arsenic, age, maternal BMI (continuous variable) and SES (using quintiles as continuous variable) were entered

Supplemental Table 8. Odds ratio (OR) and 95% confidence interval (CI) for *stunting* in children at 18, 21, and 24 months of age, in relation to quintiles of child urinary arsenic (U-As, µg/L) at 18 months of age. Data are presented for all children, boys and girls.

Quintiles of U-As at 18 months; Median (range), µg/L	18 months OR (95% CI)	21 months OR (95% CI)	24 months OR (95% CI)
All children	*n* = 1028 cases	*n* = 1069 cases	*n* = 1086 cases
Unadjusted[a]			
Q1: 12 (2.4-16)	1.00 (Reference)	1.00 (Reference)	1.00 (Reference)
Q2: 20 (16-26)	1.18 (0.91-1.54)	1.18 (0.90-1.53)	1.07 (0.82-1.39)
Q3: 34 (26-46)	1.45 (1.11-1.89)	1.18 (0.90-1.54)	1.21 (0.93-1.58)
Q4: 64 (46-96)	1.32 (1.01-1.72)	1.42 (1.09-1.86)	1.31 (1.00-1.72)
Q5: 159 (96-937)	1.04 (0.80-1.36)	1.02 (0.78-1.34)	1.08 (0.82-1.41)
Adjusted[b]			
Q1: 12 (2.4-16)	1.00 (Reference)	1.00 (Reference)	1.00 (Reference)
Q2: 20 (16-26)	1.00 (0.76-1.33)	1.02 (0.77-1.35)	0.89 (0.67-1.17)
Q3: 34 (26-46)	1.26 (0.95-1.67)	1.04 (0.79-1.38)	1.02 (0.77-1.36)
Q4: 64 (46-96)	1.09 (0.82-1.44)	1.19 (0.90-1.57)	1.06 (0.80-1.41)
Q5: 159 (96-937)	0.87 (0.65-1.15)	0.88 (0.66-1.17)	0.90 (0.67-1.19)
Boys	*n* = 556 cases	*n* = 570 cases	*n* = 584 cases
Unadjusted[a]			
Q1: 12 (2.4-16)	1.00 (Reference)	1.00 (Reference)	1.00 (Reference)
Q2: 20 (16-26)	0.88 (0.61-1.27)	0.96 (0.68-1.39)	0.88 (0.61-1.27)
Q3: 34 (26-46)	1.18 (0.82-1.69)	1.01 (0.71-1.45)	1.01 (0.71-1.45)
Q4: 64 (46-96)	1.11 (0.77-1.60)	1.14 (0.79-1.65)	1.02 (0.71-1.48)
Q5: 159 (96-937)	0.84 (0.58-1.22)	0.90 (0.62-1.31)	0.88 (0.60-1.27)
Adjusted[c]			
Q1: 12 (2.4-16)	1.00 (Reference)	1.00 (Reference)	1.00 (Reference)
Q2: 20 (16-26)	0.70 (0.47-1.03)	0.81 (0.55-1.19)	0.69 (0.47-1.03)
Q3: 34 (26-46)	0.96 (0.66-1.41)	0.82 (0.56-1.20)	0.80 (0.55-1.18)
Q4: 64 (46-96)	0.88 (0.59-1.30)	0.91 (0.62-1.35)	0.78 (0.53-1.16)
Q5: 159 (96-937)	0.71 (0.48-1.06)	0.78 (0.53-1.16)	0.71 (0.48-1.06)
Girls	*n* = 472 cases	*n* = 499 cases	*n* = 502 cases
Unadjusted[a]			
Q1: 12 (2.4-16)	1.00 (Reference)	1.00 (Reference)	1.00 (Reference)
Q2: 20 (16-26)	1.68 (1.13-2.48)	1.50 (1.02-2.22)	1.37 (0.93-2.03)
Q3: 34 (26-46)	1.85 (1.24-2.76)	1.42 (0.95-2.12)	1.52 (1.01-2.27)
Q4: 64 (46-96)	1.65 (1.12-2.43)	1.83 (1.24-2.72)	1.77 (1.20-2.63)
Q5: 159 (96-937)	1.35 (0.91-2.01)	1.22 (0.83-1.82)	1.40 (0.95-2.08)
Adjusted[c]			
Q1: 12 (2.4-16)	1.00 (Reference)	1.00 (Reference)	1.00 (Reference)
Q2: 20 (16-26)	1.49 (0.99-2.24)	1.33 (0.88-1.99)	1.17 (0.78-1.76)
Q3: 34 (26-46)	1.71 (1.13-2.58)	1.36 (0.90-2.05)	1.36 (0.90-2.07)
Q4: 64 (46-96)	1.39 (0.93-2.09)	1.58 (1.05-2.37)	1.47 (0.98-2.22)
Q5: 159 (96-937)	1.09 (0.72-1.64)	1.03 (0.68-1.55)	1.17 (0.78-1.76)

[a] Urinary arsenic (quintiles U-As exposure at 18 months) and actual age in respective age group were entered

[b] Urinary arsenic, age, sex, maternal BMI (continuous variable) and SES (using quintiles as continuous variable) were entered

[c] Urinary arsenic, age, maternal BMI (continuous variable) and SES (using quintiles as continuous variable) were entered

A)

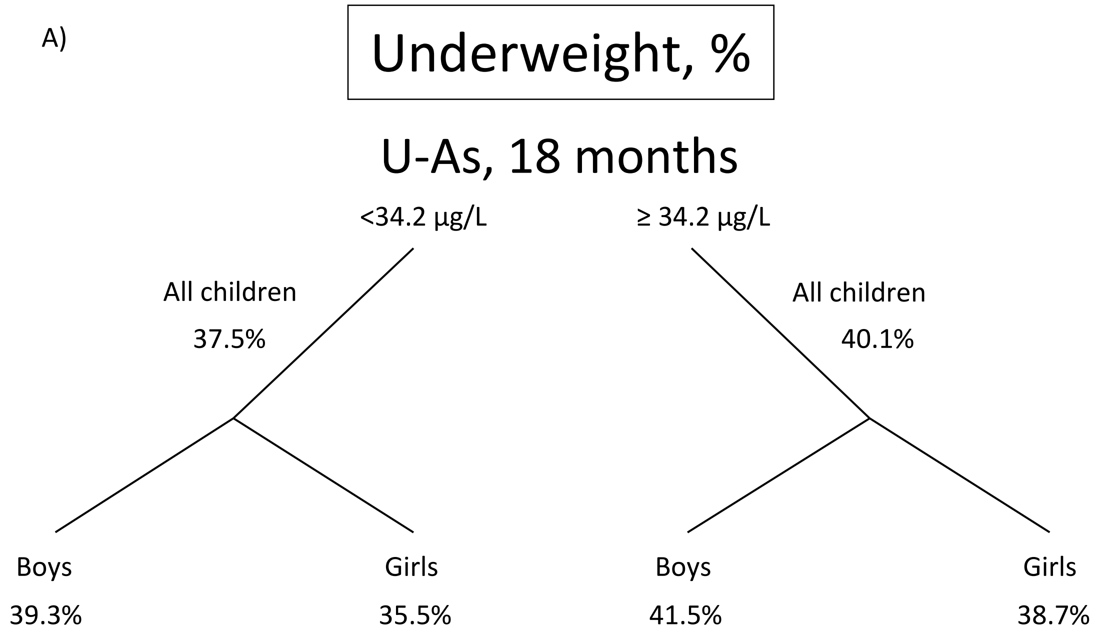

B)

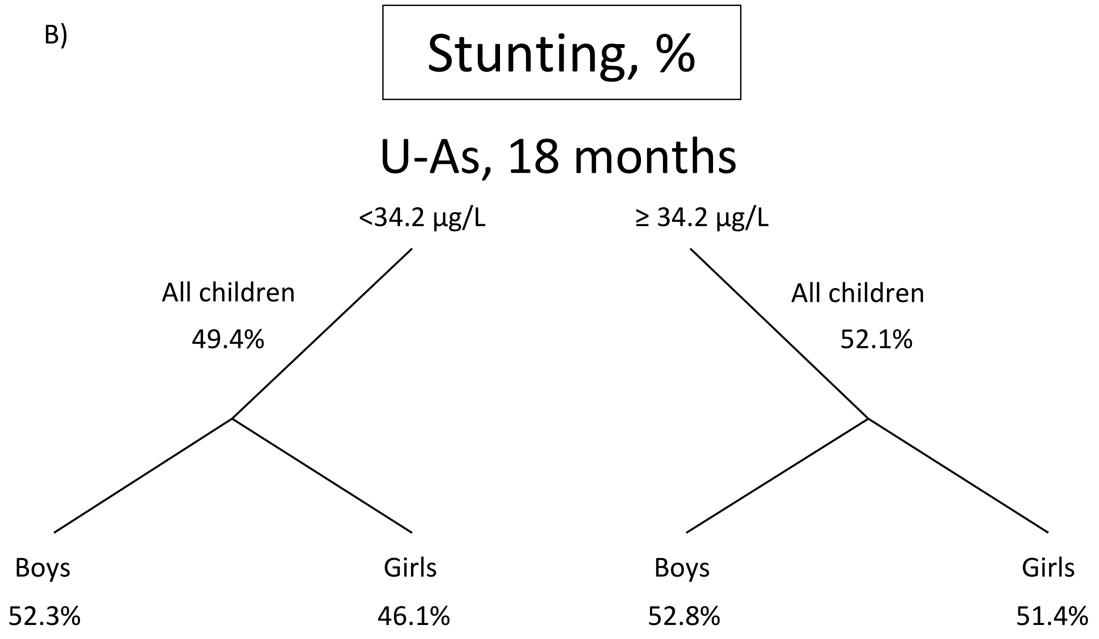

Supplemental Figure 1. Percent underweight (weight for age Z-score <-2) (A) and stunting (length for age Z-score <-2) (B) in boys and girls at 24 months of age by categorized urinary arsenic (median split, 34.2 µg/L) at 18 months.
